# Functional Characterization of Rare RAB12 Variants and Their Role in Musician’s and Other Dystonias

**DOI:** 10.3390/genes8100276

**Published:** 2017-10-18

**Authors:** Eva Hebert, Friederike Borngräber, Alexander Schmidt, Aleksandar Rakovic, Ingrid Brænne, Anne Weissbach, Jennie Hampf, Eva-Juliane Vollstedt, Leopold Größer, Susen Schaake, Michaela Müller, Humera Manzoor, Hans-Christian Jabusch, Daniel Alvarez-Fischer, Meike Kasten, Vladimir S. Kostic, Thomas Gasser, Kirsten E. Zeuner, Han-Joon Kim, Beomseok Jeon, Peter Bauer, Eckart Altenmüller, Christine Klein, Katja Lohmann

**Affiliations:** 1 Institute of Neurogenetics, University of Luebeck, 23538 Luebeck, Germany; eva.hebert@neuro.uni-luebeck.de (E.H.); friederike.borngraeber@charite.de (F.B.); alexander.schmidt@charite.de (A.S.); aleksandar.rakovic@neuro.uni-luebeck.de (A.R.); anne.weissbach@neuro.uni-luebeck.de (A.W.); jennie.hampf@neuro.uni-luebeck.de (J.H.); jule.vollstedt@neuro.uni-luebeck.de (E.-J.V.); susen.schaake@neuro.uni-luebeck.de (S.S.); humi_902@yahoo.com (H.M.); daniel.alvarez@neuro.uni-luebeck.de (D.A.-F.); meike.kasten@neuro.uni-luebeck.de (M.K.); christine.klein@neuro.uni-luebeck.de (C.K.); 2 Kurt Singer Institute for Music Physiology and Musicians’ Health, Hanns Eisler School of Music Berlin, 10595 Berlin, Germany; 3 Berlin Center for Musicians’ Medicine, Charité—University Medicine Berlin, 10117 Berlin, Germany; 4 Institute for Integrative and Experimental Genomics, University of Luebeck, 23538 Luebeck, Germany; imb9y@eservices.virginia.edu (I.B.); michaela.mueller@iieg.uni-luebeck.de (M.M.); 5 Department of Dermatology, University of Regensburg, 93053 Regensburg, Germany; Leopold.Groesser@klinik.uni-regensburg.de; 6 School of Biological Sciences, University of the Punjab, Quaid-i-Azam Campus, Lahore 54590, Pakistan; 7 Institute of Musician’s Medicine, University of Music, 01069 Dresden, Germany; Hans-Christian.Jabusch@hfmdd.de; 8 Department of Psychiatry and Psychotherapy, University of Lübeck, 23538 Lubeck, Germany; 9 Department of Neurodegenerative Diseases, Clinical Center of Serbia, 11000 Belgrade, Serbia; vladimir.s.kostic@gmail.com; 10 Department of Neurology, University of Tübingen, 72076 Tubingen, Germany; thomas.gasser@uni-tuebingen.de; 11 Department of Neurology, University of Kiel, 24105 Kiel, Germany; k.zeuner@neurologie.uni-kiel.de; 12 Department of Neurology, Movement Disorder Center, Seoul National University Hospital, Seoul 03080, Korea; cupofcoffee@daum.net (H.-J.K.); brain@snu.ac.kr (B.J.); 13 Centogene AG, 18057 Rostock, Germany; peter.bauer@centogene.com; 14 Institute of Music Physiology and Musician’s Medicine, Hanover University of Music, Drama and Media, 30175 Hanover, Germany; eckart.altenmueller@hmtm-hannover.de

**Keywords:** musician’s dystonia, RAB12, GTPase, Transferrin receptor, lysosomal degradation

## Abstract

Mutations in RAB (member of the Ras superfamily) genes are increasingly recognized as cause of a variety of disorders including neurological conditions. While musician’s dystonia (MD) and writer’s dystonia (WD) are task-specific movement disorders, other dystonias persistently affect postures as in cervical dystonia. Little is known about the underlying etiology. Next-generation sequencing revealed a rare missense variant (c.586A>G; p.Ile196Val) in *RAB12* in two of three MD/WD families. Next, we tested 916 additional dystonia patients; 512 Parkinson’s disease patients; and 461 healthy controls for *RAB12* variants and identified 10 additional carriers of rare missense changes among dystonia patients (1.1%) but only one carrier in non-dystonic individuals (0.1%; *p* = 0.005). The detected variants among index patients comprised p.Ile196Val (*n* = 6); p.Ala174Thr (*n* = 3); p.Gly13Asp; p.Ala148Thr; and p.Arg181Gln in patients with MD; cervical dystonia; or WD. Two relatives of MD patients with WD also carried p.Ile196Val. The two variants identified in MD patients (p.Ile196Val; p.Gly13Asp) were characterized on endogenous levels in patient-derived fibroblasts and in two RAB12-overexpressing cell models. The ability to hydrolyze guanosine triphosphate (GTP), so called GTPase activity, was increased in mutants compared to wildtype. Furthermore, subcellular distribution of RAB12 in mutants was altered in fibroblasts. Soluble Transferrin receptor 1 levels were reduced in the blood of all three tested p.Ile196Val carriers. In conclusion, we demonstrate an enrichment of missense changes among dystonia patients. Functional characterization revealed altered enzyme activity and lysosomal distribution in mutants suggesting a contribution of *RAB12* variants to MD and other dystonias.

## 1. Introduction

Musician’s dystonia (MD) is a task-specific movement disorder that is characterized by painless muscle incoordination or loss of voluntary motor control while the musician is playing the instrument [1,2]. It occurs in professional instrumentalists with a prevalence of 1–2% [3]. About 20% of MD patients report a positive family history, including MD or writer’s dystonia (WD) [4]. WD is another form of a task-specific dystonia involving the fingers, hand, and/or forearm. Symptoms usually appear when a person is trying to do a task that requires fine motor movements, such as writing. Of note, MD may be accompanied by additional WD [5]. Thus, MD and WD may share a molecular cause.

However, little is known about the etiology of MD and WD and both environmental and genetic factors have been discussed. Among the environmental risk factors for MD, extensively trained maximal fine-motor skills and high levels of anxiety and perfectionism have received increasing attention over the past few years [6,7,8]. On the other hand, a considerable genetic contribution is suggested by its high heritability [4,5]. In a recent genome-wide association study, an intronic variant (rs11655081) in the arylsulfatase G (*ARSG*) gene was identified as a potential genetic risk factor [9]. However, known monogenic causes of segmental and generalized dystonia including mutations in *TOR1A*, *THAP1*, and *GNAL* have been excluded as a major cause in MD [4,10,11].

RAB12, member RAS oncogene family, is part of a large family of small guanosine triphosphate (GTP)-hydrolyzing enzymes (GTPases) that play an important role in vesicle transport and trafficking within cells [12,13]. RAB12 regulates the degradation of transmembrane proteins on the membranes of different cellular compartments including the Golgi complex, endosomes and lysosomes. One well characterized target of RAB12 degradation includes the transferrin receptor 1 (TFRC) [14,15,16]. RAB12 is highly expressed in the human brain (The Human Protein Atlas, http://www.proteinatlas.org/ENSG00000206418-RAB12/tissue) and many RAB genes have been linked to neurological disorders. For instance, mutations in *RAB3* are associated with Warburg Micro syndrome [17], and functional impairments in *RAB7* have been linked to Charcot-Marie-Tooth disease Type 2B [18]. More recently, mutations in *RAB39B* were postulated as a rare cause of Parkinson’s disease [19,20].

In the present study, we initially used next-generation sequencing (NGS) in three German families with autosomal dominantly inherited MD/WD to unravel the presumably monogenic disease cause. We identified a likely pathogenic variant in the *RAB12* gene in two out of three families. Genetic screening of unrelated patients revealed two additional *RAB12* mutation carriers among MD patients. To confirm a functional effect of the identified mutations, we analyzed the consequences of these RAB12 mutations on GTPase activity, its intracellular localization, lysosomal distribution, TFRC degradation, and autophagy. Additional *RAB12* variants were found in other dystonia patients but were largely absent in the investigated controls as well as in publically available databases.

## 2. Materials and Methods

### 2.1. Subjects

**Figure 1:**
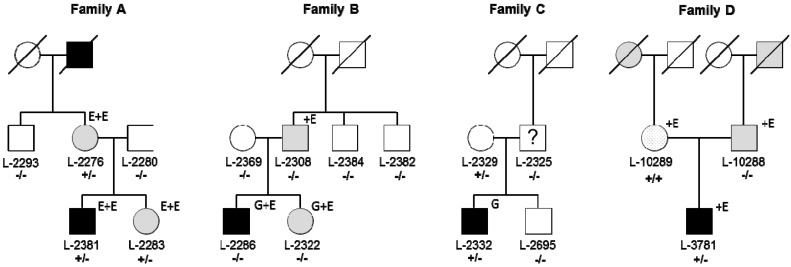
Pedigrees of four musician´s dystonia (MD) families. Black symbols represent individuals with MD, gray symbols mark individuals with WD, the dotted filled symbol indicates an individual with possibly mild, generalized dystonia, and the question mark points to an individual that was initially reported to have dystonia which however was not confirmed upon re-evaluation by two neurologists. L-2329 did not agree to be neurologically examined. A photograph of L-10289 illustrating her dystonic postures is shown in Figure S1. Patients of Family A were exome sequenced at Atlas Biolabs (superscript “E”) and three patients of Families B and C underwent genome sequencing at Complete Genomics (superscript “G”). Additional exome sequencing was performed for individuals of Families A, B, and D at Centogene (superscript“+E”). Mutational status for the p.Ile196Val mutation in RAB12 is indicated: +/+ for homozygous carriers; +/− for heterozygous carriers; −/− for non-carriers. For individuals for whom DNA was available a sample ID number (L code) is shown. Dashed symbols represent deceased family members.

The study was approved by the ethics committee at the University of Lübeck (Lübeck, Germany, No 04-180 from 1 July 2005). All participants gave written informed consent. We included a total of 1906 subjects (Table 1) comprising 241 professional musicians diagnosed with MD, 14 relatives from four MD families (Figure 1), 74 WD patients, 604 other dystonia patients (Table S1), 512 patients with Parkinson’s disease (PD), and 461 healthy controls from the population-based control cohort EPIPARK from Lübeck (Germany) [21]. All subjects were of European origin (mainly German) with the exception of 86 dystonia patients from South Korea. Three of the MD patients and their families (Families A–C, Figure 1) were previously reported [4,22] and originated from Germany. The diagnostic work-up of MD patients included a complete neurological examination and visual inspection while patients were playing their instruments. All other patients were diagnosed by movement disorder specialists in Lübeck, Kiel, Tübingen (Germany), Seoul (South Korea), and Belgrade (Serbia).

### 2.2. Mutation Screening

We initially performed exome or genome sequencing in 2011/2012 in six patients from three families (Figure 1). Specifically, genome sequencing was carried out in three patients of Families B and C (Complete Genomics, Mountain View, AB, Canada) while three patients of Family A were exome sequenced on an Illumina Genome Analyzer (Atlas Biolabs, Berlin, Germany). Variant calling and annotation were performed by Complete Genomics and Atlas Biolabs, respectively. Detected variants were filtered (a) to be exonic or splicing; (b) to affect amino acid sequence (synonymous variants were discarded); (c) to be rare, with a known frequency < 1% in the database for single nucleotide polymorphisms (dbSNP132, http://www.ncbi.nlm.nih.gov/projects/SNP/ snp_summary.cgi?build_id=132); and (d) to be shared among definitely affected members within a family (Table S2). We further hypothesized that at least two of the families shared a mutation in the same gene. Candidate variants were validated by Sanger sequencing. All available family members were tested for segregation using Sanger sequencing.

We recently (in 2016) repeated exome sequencing in Family A (3 affected) and B (3 affected), and also included Family D (patient-parent trio) at Centogene, Rostock, Germany using an Illumina HiSeq 2000 machine and an in-house-annotation pipeline. In each of these families, we filtered for rare, protein-changing variants that were shared by all affected within a given family (Table S3).

Based on the NGS analyses, a rare variant in *RAB12* was the only plausible candidate. Next, we used Sanger sequencing to screen Exons 2 to 6 of *RAB12* in 238 unrelated German MD patients, 54 WD patients, 378 patients with different forms of dystonia, and 461 unrelated healthy subjects. Exon 1, which has a high GC content of 78% and thus was difficult to amplify, was tested in all MD and WD patients as well as in 170 healthy controls. Primer sequences are shown in Table S4.

Furthermore, all exons and exon/intron boundaries of *RAB12* were included in an NGS-based gene panel analysis (Centogene, Rostock, Germany) that was performed for another 246 dystonia and 512 PD patients.

### 2.3. cDNA Analysis

A synonymous variant (c.276A>G, p.Arg92=) in Exon 2 of *RAB12* that was predicted by MutationTaster to affect splicing of *RAB12* was investigated on the RNA level. For this, RNA was extracted from a blood sample of a carrier using the QIAmp RNA Extraction Kit (QIAGEN, Germantown, MD, USA). Oligo-dT-Nucleotides of the Maxima First Strand cDNA Synthesis Kit (ThermoFisher, Waltham, MA, USA) served as primers to synthesize the complementary DNA (cDNA) by use of reverse transcriptase. PCR was performed with primers in Exons 1 and 4 (Table S4) and the respective product was inspected for its size and Sanger sequenced.

### 2.4. Plasmid and Viral Construction

The complete coding sequence of *RAB12* (RefSeq: NM_001025300.2) was subcloned into a pcDNA vector containing a FLAG ^TM^-tag upstream of *RAB12*. The FLAG-tagged *RAB12* sequence was then introduced into a lentiviral expression vector containing a puromycin resistance cassette. The mutations (c.38G>A, p.Gly13Asp and c.586A>G, p.Ile196Val) were introduced by site-directed mutagenesis (QuikChangeII, Stratagene, Cedar Creek, TX, USA, for primers see Table S4). Sequences of all *RAB12* constructs were verified by Sanger sequencing. For lentiviral production, Human Embryonic Kidney (HEK293T) cells were transfected with vectors containing vsv-g envelopes, pCMV delta R8.2, and expression vectors with the *RAB12* constructs using FuGENE HD transfection reagent (Promega, Madison, WI, USA). The virus was harvested from the supernatant by ultracentrifugation and the titer was determined using the RETRO-TEK HIV p24 Antigen ELISA (enzyme-linked immunosorbent assay) (Zeptometrix, Buffalo, NY, USA).

### 2.5. Cell Culture and Stable Transfection

Skin biopsies were collected from two MD patients carrying the Ile196Val mutation and two healthy controls. Fibroblasts and SH-SY5Y cells were cultivated in Dulbecco’s modified eagle medium supplemented with 10% fetal bovine serum and 1% penicillin/streptomycin (all medium components were provided by PAA Laboratories, Pasching, Upper Austria, Austria). The cells were incubated at 37 °C and 5% CO_2_ in a humidified atmosphere. Fibroblasts of the MD patients were used to study effects of mutations in endogenously expressed RAB12. For overexpression studies, SH-SY5Y cells and fibroblasts from a healthy control subject were stably transfected with lentivirus (multiplicity of infection = 5) containing expression vectors with *RAB12* wildtype (WT) and mutated cDNA sequences (c.38G>A, p.Gly13Asp and c.586A>G, p.Ile196Val). Selection of transfected cells was performed with 2 µg/mL puromycin (Sigma, St. Louis, MO, USA).

### 2.6. GTPase Assay

Proteins from SH-SY5Y cells stably expressing RAB12 WT and mutated proteins were extracted with an isotonic lysis buffer (15 mM Tris/HCl pH 7.4, 150 mM NaCl, 1% NP-40, 1 mM EDTA (Ethylendiamin-tetraacetat) protease inhibitor cocktail (Roche, Basel, Switzerland)) and subsequently centrifuged at 4 °C at 16,000× *g* for 20 min. Protein concentration was measured using the Dc Protein Assay (BioRad, Hercules, CA, USA). Whole protein extracts were used for the measurement of GTPase activity because effector proteins of RAB12, except for the Guanine nucleotide exchange factor DENND3 (DENN domain containing 3) [23], are largely unknown. The rate of inorganic phosphate that was newly produced within one hour of incubation was determined with the ATPase/GTPase Activity Assay Kit (Sigma), which was used according to the manufacturer’s protocol. Absorption was measured at 620 nm. The specific GTPase activity was calculated and data were normalized for the GTPase activity in RAB12 WT in each experiment. Means of four independent experiments were calculated.

### 2.7. RAB12 Protein Structure Modeling and Molecular Dynamics Simulations

The X-ray structure of inactive, guanosine diphosphate (GDP)-bound WT RAB12 (Protein Data Bank [PDB] ID: 2IL1, www.rcsb.org) was prepared using the Protein Preparation workflow (Schrödinger Suite 2014-2 Protein Preparation Wizard; Epik version 2.8; Impact version 6.3; Prime version 3.6, Schrödinger, LLC, New York, NY, 2014) [24]. Inactive p.Ile196Val-RAB12 was modeled with Schrödinger Prime (version 3.6, Schrödinger, LLC, New York, NY, 2014) based on the 2IL1-X-ray. Active, GTP-bound RAB12 models were built using the RAB1A X-ray structure (Protein Data Bank (PDB) ID: 3TKL [25], chain A, resolution: 2.18 Å) and the homology-modeling module of Schrödinger Prime (version 3.6) with default settings. Non-template loops were refined by loop refinement (Prime, version 3.6). Additionally, the models were optimized employing the OPLS2005 [26] force field for energy minimization. Validation of the homology models was done using Prosa2003 [27] and PROCHECK. [28] All molecular dynamics simulations were performed using the Desmond package (Desmond Molecular Dynamics System, version 3.8, D. E. Shaw Research, New York, NY, USA, 2014) [29] and the OPLS 2005 force field [26]. Prepared X-ray structures and homology models of RAB12 were used as starting structures; each system was solvated in an orthorhombic box of TIP3P (transferable intermolecular potential with 3 points)-modeled water molecules [30]. The simulations were carried out with the default protocol provided in Desmond. Molecular dynamics simulations were performed at 300 K and 1bar for either 10 ns or 20 ns, if not stable after 10 ns simulation.

### 2.8. Immunostaining

For immunostaining, patient-derived fibroblasts, control fibroblasts, and fibroblasts stably expressing WT and mutant forms of RAB12 were cultured on glass cover slips in a 24-well plate. Cells were fixed with 4% paraformaldehyde (Sigma) and FLAG-tagged RAB12 proteins were detected with a primary antibody raised against FLAG (1:1000, rabbit, Cell Signaling, Cambridge, UK). For detection of lysosomes and TFRC, monoclonal antibodies raised against LAMP-1 (lysosomal associated membrane protein 1) as a lysosomal marker (1:200, mouse, Santa Cruz, Dallas, TX, USA) and TFRC (1:300, mouse, Invitrogen, Carlsbad, CA, USA) were used. The Endoplasmic reticulum (ER) and the Golgi apparatus were detected with monoclonal antibodies raised against PDI (1:500, mouse, Abcam, Cambridge, UK) and 58K Golgi protein (1:50,000, mouse, Abcam), respectively. Alexa fluor 488 and Alexa fluor 568 coupled secondary antibodies (1:400, goat, Life Technologies, Carlsbad, CA, USA) were utilized for visualization. Cell nuclei were stained with 1 µg/mL DAPI in DAPI Fluoromount G mounting medium (Southern Biotech, Birmingham, AL, USA). Images were analyzed with a Zeiss Axiovert 200 M microscope (Zeiss, Oberkochen, Germany) with ApoTome and Axiovision Rel 4.8 software (Zeiss, Oberkochen, Germany). For each of the three constructs (1 WT, 2 mutants), we randomly chose areas for analysis. Within these areas, all cells with intact nuclei (in total 110 cells per construct) were analyzed and assigned to one of three groups according to their properties: (a) RAB12 and lysosomes were uniformly distributed in the cytosol; (b) RAB12 and lysosomes were located in the perinuclear region and in large parts of the cytosol; (c) RAB12 and lysosomes accumulated exclusively in the perinuclear region.

### 2.9. Autophagy Inhibition and TFRC Degradation

SH-SY5Y cells stably expressing mutant and WT RAB12 were treated with the lysosomal inhibitor Bafilomycin A1 (3 nM, Sigma) for 24 h. Cells were detached using Accutase (PAA laboratories, Pasching, Austria) and proteins were extracted with RIPA buffer (50 mM Tris-HCl, pH 7.6, 150 mM NaCl, 1% deoxycholate (DOC), 1% NP-40 and 0.1% sodium dodecyl sulfate (SDS)). After centrifugation at 4 °C at 16,000× *g* for 20 min, the proteins of the supernatant were used for Western blot analysis. Protein concentrations were determined utilizing Dc Protein Assay (BioRad) and 10 µg of the proteins were loaded on NuPAGE 4–12% Bis-Tris protein gels (Life technologies). Proteins were then transferred to a nitrocellulose membrane (Protran, AnalytikJena, Jena, Germany) and primary antibodies raised against FLAG (1:1 × 10^7^, mouse, Sigma), TFRC (1:20,000, mouse, Invitrogen), p62 (1:1000, rabbit, Cell Signaling), LC3 (1:4000, rabbit, Novus, Wiesbaden Nordenstadt, Germany), and β-actin (1:1 × 10^6^, mouse, Cell Signaling) were used. The signal intensities of TFRC, p62, β-actin, and LC3 II (light chain 3, also known as microtubule associated protein 1 light chain 3 alpha) were analyzed densitometrically with the Totallab 100 v2006 software. For calculations of TFRC degradation, the protein bands of bafilomycin-treated cells were set to 100%.

### 2.10. Endogenous TFRC Levels in Patients’ Blood

To test for the concentration of soluble TFRC levels in the blood of available patients from Family A (L-2283, L-2381, L-2276), we used routine blood count measurements from local medical laboratories.

### 2.11. Statistical Analysis

To compare frequencies of diseased and healthy *RAB12* variant carriers, Chi-square test (comparison with frequencies in about 60,000 individuals from the Exome Aggregation Consortium (ExAC) at http://exac.broadinstitute.org/) and Fisher’s exact test (comparison with our control individuals) were performed. All statistical tests related to the functional assays were performed with Graph Pad Prism 6. One-way analysis of variance (ANOVA) with Bonferroni’s post-hoc test was carried out to analyze the effect of *RAB12* mutations on the GTPase activity, TFRC degradation, and the activation of autophagy. A Chi-square test was used for comparison of the cellular distribution of RAB12 and LAMP-1 in fibroblasts stably expressing WT or mutant *RAB12*. Likewise, for comparison of LAMP-1 distribution in patient and control fibroblasts, a Chi-square test was utilized.

## 3. Results

### 3.1. Identification of RAB12 Mutations in Patients with Musician’S Dystonia

**Figure 2:**
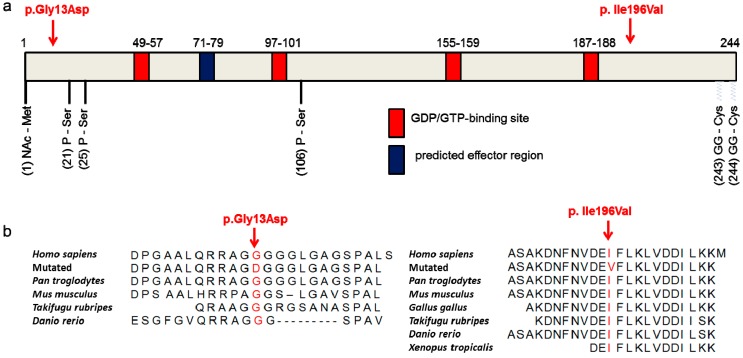

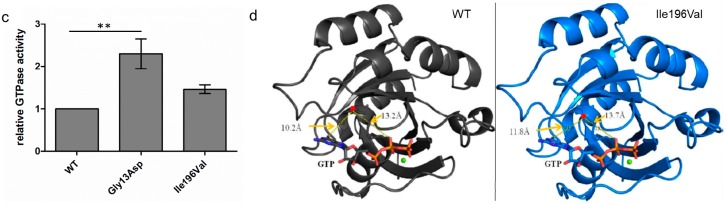
RAB12 and functionally investigated variants. (**a**) Schematic view of the RAB12 protein with the identified mutations p.Gly13Asp and p.Ile196Val in MD patients (red arrows), the guanosine diphosphate/guanosine triphosphate (GDP/GTP)-binding sites (red), the predicted effector region (blue), and the posttranslational modifications on amino acid positions 1 (N-acetylation), 21, 25, 106 (phosphorylations), 243, and 244 (geranylgeranylations) are indicated. RefSeq: NM_001025300.2, NP_001020471; (**b**) The Glycine at position 13 (left panel, red) and the Isoleucine at position 196 (right panel, red) are conserved across different species; (**c**) Proteins of transfected SH-SY5Y cells were incubated with GTP for 1 h and PO_4_^3−^ production was measured. GTPase activity of mutant RAB12 expressing cells was normalized for GTPase activity of wild-type (WT) RAB12 expressing cells. The bars indicate the means ± standard error of mean, One-way analysis of variance (ANOVA) with Bonferroni’s post-hoc test. ** *p* < 0.01, *n* = 4; (**d**) Homology model of active/GTP-bound RAB12 in ribbon representation. WT is presented in black (left model) and p.Ile196Val in blue (right model). Magnesium ions are shown as green spheres, amino acid residue 196 is marked by red points, and GTP is indicated by sticks. The figures were generated with PyMOL (PyMOL Molecular Graphics System, Version 1.7.0 Schrödinger, LLC, New York, NY, USA).

Next-generation sequencing analyses in 2011/2012 revealed about 25,000 variants per exome and approximately 3.5 million variants per genome (Table S2). Filtering for rare, exonic and splicing as well as protein-changing variants shared among affected individuals within families, revealed 83, 509, and 1023 changes in Families A, B, and C, respectively. While there was no gene with rare, protein-changing variants in affected individuals of all three families, the same rare missense change in *RAB12* (c.586A>G, p.Ile196Val, rs143888944) was found among the four affected patients of Families A and C (Figure 1 and Figure 2a). This variant was also detected in the presumably unaffected mother (L-2329) of the index patient in Family C. She is a 76-year old, retired secretary who did not play a musical instrument. She did not report any dystonic symptoms on the telephone-based Beth Israel Medical Center Dystonia Screen (BIDS) but could not be neurologically examined [4].

This *RAB12* variant was not detected in 461 ethnically matched controls and has been reported in GnomAD in the heterozygous state only with a frequency of 0.0003. The p.Ile196Val substitution is highly conserved (Figure 2b) and predicted to be damaging by MutPred (http://mutpred.mutdb.org/) and MutationTaster (www.mutationtaster.org), and received a Combined Annotation Dependent Depletion (CADD) score of 16.5 (http://cadd.gs.washington.edu/) (Table 2).

Next, we searched for genetic variations of *RAB12* in other MD patients. Sequencing of all six coding exons in 238 unrelated MD patients of European ancestry revealed two additional carriers of rare missense variants (p.Ile196Val, p.Gly13Asp) as well as one carrier of a synonymous change (p.Arg92=, Table 2 and Table 3). The p.Ile196Val substitution was detected in another 39-year old German MD patient (Patient D, Figure 1). The patient first noticed embouchure problems when playing the trumpet at the age of 17 years. He changed his instrument and started to study the tuba. At the age of 21 years, he also developed embouchure dystonia on the tuba and had to stop his musical career. His father had WD (age at onset: 27 years) and the maternal grandmother as well as the paternal grandfather were both reported to also suffer from writing problems (Figure 1). Segregation analysis revealed that the mildly affected mother of Patient D carried the p.Ile196Val variant in the homozygous state. Upon neurological examination and based on an unblinded as well as a blinded video review by three independent movement disorder specialists, she displayed several mild motor signs. These included dystonic posturing of her right hand with flexion of the third to fifth finger and of the thumb and extension of the index finger, perioral dyskinesia entrained with hand and finger movements, slowness of finger and hand movements bilaterally, more pronounced on the right-hand side and accompanied by curling of the third to fifth finger, as well as a 15-degree tilt to the right of her trunk in keeping with mild axial dystonia (Figure S1). The father was carrier of two WT alleles. He also tested negative for the GAG deletion in *TOR1A* (NM_000113.2: c.904_906delGAG, p.Glu303del) and negative for mutations in other dystonia-related genes [31] by exome sequencing.

The second missense variant (c.38G>A, p.Gly13Asp) was found in a 36-year old percussionist suffering from MD (Patient E, Figure 2a,b). He developed task-specific dystonia of his right leg when playing the percussion at the age of 25 years.

The synonymous variant was located near the acceptor splice site of Exon 2 (c.276A>G, p.Arg92=) and detected in a 39-year old MD patient. The variant was predicted to affect splicing by MutationTaster. However, analysis of mRNA expression revealed only one transcript and sequencing of the cDNA confirmed the presence of both alleles, indicating that the variant does not impair splicing (data not shown).

Recent exome sequencing of Families A, B, and D with good coverage (mean coverage depth: 120×, with approximately 95% of bases covered at >20×) excluded known dystonia genes and confirmed *RAB12* as the only plausible candidate (Table S5). Except for variants in *MUC4* in Families B and D, none of the candidate genes from an individual family were found in a second family. *MUC4* encodes a mucin that is expressed in epithelial cells and does not represent a convincing candidate for a neurological disorder. In Family A, B, and D, we identified 60, 46, and 80 individual, rare, protein-changing variants shared among the affected family members (Table S5).

### 3.2. RAB12 Variants Are More Frequent in Dystonia Patients

The detection of four MD patients carrying rare missense variants in *RAB12* was followed by genetic analysis of additional patients and controls. Among 74 unrelated WD patients, we found another carrier of p.Ile196Val (L-9497) and two patients with another substitution (c.520G>A, p.Ala174Thr, L-11086, L-11092). Of note, the first symptoms in L-11086 occurred in his left hand after prolonged clarinet playing over two months for more than 10 hours in an army band, indicating that he actually suffers from MD. Screening of 604 non-MD/WD dystonia patients revealed a total of five heterozygous carriers of rare missense variants in RAB12 including p.Ile196Val (*n* = 2), p.Ala174Thr, c.442G>A (p.Ala148Thr), and c.542G>A (p.Arg181Gln, rs371288995) (Table 2 and Table 3).

Among 461 healthy controls, we found one carrier of a rare missense variant (p.Asp131Leu) and two carriers of synonymous base pair substitutions (p.Arg92=, p.Pro231=) (Table 2). While p.Arg92= did not affect splicing (see above), an RNA sample of the carrier of the p.Pro231= variant was not available. Both synonymous variants received a relatively low CADD score (<11) and activation of a cryptic splice site was not predicted using a splice site prediction tool (http://www.fruitfly.org/seq_tools/splice.html, donor and acceptor score cutoff: 0.1). In 512 PD patients that were screened using a GenePanel and served as controls to test for disease specificity of the *RAB12* variants, we did not detect any rare sequence change (Table 2).

### 3.3. GTPase Activity Seems to Be Elevated in RAB12 Mutants

We aimed to test whether the RAB12 missense variants identified in MD patients (p.Gly13Asp, p.Ile196Val) have an impact on the enzymatic activity of the small GTPase RAB12. To compare the GTPase activity between SH-SY5Y cells stably expressing FLAG-tagged WT, p.Gly13Asp, and p.Ile196Val forms of RAB12, proteins were isolated in a native buffer and GTP hydrolysis was performed for one hour. Colorimetric measurements in four independent experiments revealed that GTP hydrolysis in FLAG-RAB12 p.Gly13Asp expressing SH-SY5Y cells was significantly increased compared to SH-SY5Y cells expressing FLAG-RAB12 WT (2.3-fold, *p* = 0.0065, Figure 2c). The GTP hydrolysis in FLAG-RAB12 p.Ile196Val expressing SH-SY5Y cells seemed also to be increased (1.4-fold) but the effect did not reach significance. Both mutations are located outside of the predicted GDP/GTP binding sites (49–57, 97–101, 155–159, and 187–188, www.uniprot.org, Figure 2a) and the predicted effector protein binding site (amino acids 71–79, www.uniprot.org).

### 3.4. The p.Ile196Val Mutation Does Not Change the Secondary and Tertiary Structure of RAB12 In Silico

To investigate the impact of RAB12 mutations on the protein structure, in silico modeling was performed. RAB proteins switch between a GDP- (inactive) and a GTP-bound (active) conformation and these two protein states differ in the conformation of the switch regions [32]. A RAB12 X-ray structure (PDB ID: 2IL1, resolution: 2.1 Å) in complex with GDP representing the inactive form is publically available. Amino acid residue Gly13 is missing in this crystal structure and could not be modeled. Since no 3D structure of RAB12 in its active conformation is available, homology models of active, GTP-bound WT- and p.Ile196Val-RAB12 were built. The human RAB1A X-ray structure (PDB ID: 3TKL [25], chain A, resolution: 2.18 Å), representing the active form, was used as template (50% sequence identity with RAB12 and covering Arg37-Met208). Homology models of active RAB12 WT and p.Ile196Val were generated (Figure 2d). Gly13Asp was not covered by the template and thus could not be modeled. Residue 196 is located in an α-helix about 10 Å away from GDP/GTP and its binding site. Using these models, there was no major effect on GDP/GTP binding or protein structure comparing the molecular dynamics simulations of WT- and p.Ile196Val-RAB12 (inactive and active, data not shown).

### 3.5. RAB12 Mutations Alter the Subcellular Localization of RAB12 and Lysosomes

**Figure 3:**
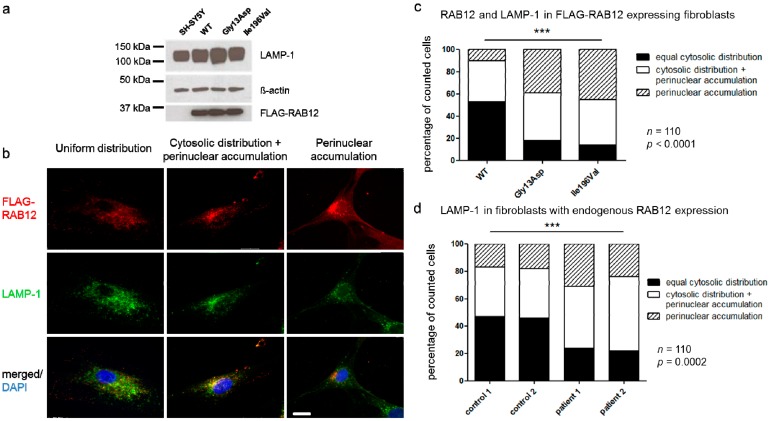
RAB12 mutations changed the subcellular localization of RAB12 proteins and lysosomes. (**a**) Ectopical protein expression of FLAG-RAB12 and expression of the lysosome-associated protein LAMP-1 (lysosomal associated membrane protein 1) is equal in RAB12 WT and mutant SH-SY5Y cells. All protein signals were detected on the same membrane. Blots were cropped for clarity but no bands were removed. Original blots are shown in the supplementary material (Figure S5); (**b**) In WT and mutated RAB12-overexpressing cells, three different patterns of RAB12 distribution were observed and categorized. Proportions of cells revealed RAB12 (red) that was evenly distributed in the cytoplasm (upper left panel), that was distributed in the cytoplasm and accumulated in close proximity to the nucleus (upper middle panel), or that accumulated exclusively in the perinuclear region of the fibroblast (upper right panel). FLAG-RAB12 and LAMP-1 (green) colocalized in the cytoplasm (lower panels). Nuclei were stained with DAPI (blue). Scale bar: 20 µm; (**c**) 110 randomly chosen fibroblasts with each construct were categorized and cellular localization of FLAG-RAB12 and LAMP-1 was quantified. Chi-square test: *p* < 0.0001; (**d**) 110 randomly chosen fibroblasts of two MD patients (p.Ile196Val) and two healthy controls were categorized and cellular localization of LAMP-1 was quantified. Chi-square test: *p* = 0.0002.

An increase in active/GTP-bound RAB12 may result in altered RAB12 function. In a first step, we confirmed that transfected SHSY-5Y cell lines expressed similar levels of mutant and WT RAB12 as examined by Western blotting. Further, the RAB12 mutants had no effect on the levels of the lysosomal marker LAMP-1 (Figure 3a). To investigate the cellular localization of mutant (p.Gly13Asp, p.Ile196Val) and WT RAB12, immunofluorescent staining was performed in fibroblasts expressing FLAG-tagged mutant and WT RAB12. We observed in each line different patterns of RAB12 distribution that were classified as (a) uniform distribution of RAB12 throughout the cytoplasm (example see Figure 3b, upper left panel); (b) accumulation of RAB12 in the perinuclear region (Figure 3b, upper right panel); and (c) a combination of cytoplasmic and perinuclear localization (Figure 3b, upper middle panel). While WT RAB12 demonstrated uniform cytoplasmic localization in 53% of cells, this type of distribution was only present in 18% of cells with p.Gly13Asp and 15% of cells with p.Ile196Val overexpression (Figure 3c). Accordingly, the percentage of cells with exclusively perinuclear accumulation of RAB12 was lower in fibroblasts expressing WT (10%) compared to those expressing mutant proteins (p.Gly13Asp: 39%, p.Ile196Val: 45%, *p* < 0.0001).

RAB12 has previously been shown to colocalize with lysosomes [15]. Here, we evaluated lysosomal localization of mutant and WT RAB12 by immunocytochemistry using the lysosomal marker LAMP-1 and an anti-FLAG antibody (since there is no human RAB12 antibody commercially available for immunocytochemistry). We observed lysosomal localization of WT and both mutant forms of RAB12 (Figure 3b, lower panels). Of note, immunofluorescence experiments revealed no colocalization of FLAG-RAB12 WT and mutated proteins with the ER or the Golgi apparatus (data not shown). In the patient-derived fibroblasts expressing endogenous levels of mutant RAB12, the number of cells with uniformly distributed lysosomes was reduced by about 50% compared to control fibroblasts (Figure 3d). There was also an increase in the fraction of cells with predominant perinuclear accumulation in the patient fibroblasts compared to controls (controls: 17% and 18%, patients: 24% and 31%, *p* = 0.0002).

### 3.6. Soluble TFRC Levels in Patients’ Blood Were Reduced

It has been reported that RAB12 regulates the degradation of TFRC [15]. All three tested patients with the p.Ile196Val mutation showed reduced soluble TFRC levels in blood (Table 4). While in both female WD patients soluble TFRC levels at 0.7 and 0.8 mg/L were only slightly below the reference range (0.83–1.76 mg/L), levels in the male MD patient at 1.7 mg/L were more clearly below the reference range (2.2–4.99 mg/L). Except for the Transferrin level which was slightly decreased in the male patient (2.3 g/L, ref: 2.50–3.80g/L), all other parameters reflecting iron metabolism were in the normal range in all three patients (Table 4).

### 3.7. Colocalization of RAB12 and TFRC and Degradation of TFRC Was Unchanged in RAB12 Mutants

RAB12 colocalizes with the transmembrane receptor TFRC, which is possibly taken up by endocytosis and recycled back to the cell membrane via recycling endosomes or degraded via the lysosomal pathway [15]. We confirmed colocalization of FLAG-RAB12 and TFRC in fibroblasts overexpressing WT RAB12 and demonstrated colocalization also for mutated RAB12 (p.Gly13Asp, p.Ile196Val, Figure S2). Due to enhanced GTPase activity of RAB12 mutants, lysosomal degradation of TFRC might be altered. To exhibit physiological receptor function, TFRC forms dimers. Therefore, we investigated the degradation of dimeric TFRC in WT and mutated FLAG-RAB12-expressing fibroblasts and SH-SY5Y cells, and in patient fibroblasts. Inhibition of lysosomal acidification by treatment with Bafilomycin A1 for 24 h led to accumulation of dimeric TFRC in all three cellular models in RAB12 WT and mutant cell lines (Figure S3) but no difference on TFRC degradation was observed dependent on the RAB12 form. After 24 h, about 20–40% of TFRC were degraded (Figure S3).

### 3.8. Autophagy Is Not Impaired in SH-SY5Y Cells Overexpressing RAB12 Mutants

Since we observed an altered distribution of lysosomes in cells expressing mutant RAB12 and as RAB12 regulates the degradation of the amino acid transporter SLC36A4 (solute carrier family 36 member 4, also known as PAT4) [16], a regulator protein of mTOR (mammalian/mechanistic target of rapamycin complex 1)-dependent autophagy, we hypothesized that autophagy might be disturbed in RAB12 mutants. To assess the autophagy flux, we analyzed conversion of the autophagy marker MAP1LC3A (LC3) by Western Blot. Upon activation of autophagy, LC3 translocates from the cytosol to the autophagic membrane and converts from its ‘inactive’ form LC3-I into an autophagy-related form LC3-II [33]. Treatment of SH-SY5Y cells expressing FLAG-RAB12 WT and mutated RAB12 with 3 nM Bafilomycin A1 for 24 h, raised the levels of lipidated active LC3-II protein (Figure S4a), which is a component of the autophagosome membrane. The LC3-II levels relative to ß-actin levels were calculated as a measurement of activation of autophagy and were demonstrated to be not significantly elevated in mutated FLAG-RAB12 expressing SH-SY5Y cells (p.Gly13Asp: +1.2-fold; p.Ile196Val: +1.1-fold) compared to the FLAG-RAB12 WT expressing cells (Figure S4b, *p* = 0.2). Protein levels of p62, a sensor for toxic cellular waste that accumulates when autophagy is impaired, were not altered in SH-SY5Y cells expressing mutant FLAG-RAB12 compared to WT FLAG-RAB12 proteins (Figure 3c,d).

## 4. Discussion

We here report an enrichment of rare missense variants in dystonia patients (12/919, 1.3%), particularly in patients with MD (5/242, 2.1%) compared to healthy controls (1/461, 0.2%) and PD patients (0/512). Specifically, we detected the p.Ile196Val substitution in 3 of 241 patients of our specifically recruited MD patients and in two relatives with WD as well as in one unrelated WD patient. Rare missense variants in two additional MD patients included p.Gly13Asp and p.Ala174Thr. The latter change was found in a patient of South Korean origin who was initially grouped among the WD patients but co-incidentally also suffered from MD. Ala174Thr was also found in a patient with segmental dystonia including WD. A total of 5 additional carriers of rare variants were found among 604 patients with other forms of dystonia (cervical dystonia) including two carriers of p.Ile196Val, as well as one carrier each of a p.Ala174Thr, p.Ala148Thr, or p.Arg181Gln substitution. There were no family members available to test for segregation but all missense changes received a CADD score > 15 and were extremely rare (<0.0006) in public databases, including GnomAD (genome aggregation database, Table 2).

For statistical analysis, we compared the number of missense variant carriers in dystonia patients (10/916, not including the initial families) and the non-dystonic subjects (1/973) which yielded a significant *p*-value of 0.005 (two-tailed Fisher´s exact test). Although this included three South Korean dystonia patients for whom we did not have ethnically matched controls, the difference for our overall study remains significant even when focusing on Caucasians by taking out the 86 South Korean patients (7/830 vs. 1/973, *p* = 0.0278 [Fisher´s exact test]). Of note, the Ala174Thr that we found in three South Korean patients is found almost exclusively in the East Asian population with a carrier frequency of 120/18864 in GnomAD (http://gnomad.broadinstitute.org/variant/18-8636254-G-A) and seems to be enriched in dystonia patients (albeit not statistically significant; 3.5% vs. 1.3%).

*RAB12* is located on chromosome 18p11.22 and the encoded protein belongs to a large family of small GTPases, which play an important role in vesicle transport and trafficking within cells [12,13]. RAB12 is reported to be located on the membranes of different cellular compartments, including the Golgi complex, endosomes, and lysosomes where it regulates degradation of transmembrane proteins [14,15,16]. Of note, we here demonstrated colocalization with a lysosomal marker but not with the ER or the Golgi complex.

Our in vitro studies focused on the two initially identified missense variants (p.Gly13Asp and p.Ile196Val) in RAB12 among MD patients and revealed functional alterations, indicating several lines of support for a possible pathogenic role of these substitutions in RAB12. This includes an increased GTPase activity that was observed for RAB12 mutant cells despite the localization of the mutations outside of the reported GTP-binding sites of RAB12. This elevated enzymatic activity could not be explained by 3D modeling and simulations of p.Ile196Val. Of note, the Gly13Asp variant that showed a >2-fold increase in GTPase activity, could not be modeled due to lack of a suitable template. Considering the same genetic background in RAB12 overexpressing SH-SY5Y cells, an indirect effect of RAB12 mutations on GTP hydrolysis is also possible. Interestingly, we observed perinuclear accumulation of RAB12 and lysosomes in cells with RAB12 mutations, which may be related to the altered GTPase activity. This idea is supported by recently published findings showing perinuclear clustering of constitutively active RAB12 in RBL-2H3 (rat basophilic leukemia) cells [34].

Furthermore, RAB12 is thought to be involved in iron metabolism by regulating the degradation of TFRC [15]. Cells take up transferrin-bound TFRC via receptor-mediated endocytosis. This mechanism is essential for iron uptake in neural tissue [35]. Of note, iron deficiency is implicated in some types of neurodegeneration [36]. Furthermore, in several genetic forms of neurodegeneration with brain iron accumulation (NBIA), dystonia (DYT) is a prominent feature of the disease such as in NBIA/DYT-PANK2 [37], NBIA/DYT/PARK-PLA2G6 [38], NBIA/DYT/PARK-CP [39], and NBIA/DYT-DCAF17 [31,40]. Interestingly, in all three tested patients from Family A, the levels of soluble blood TFRC were reduced.

Besides the reported regulatory impact of RAB12 on TFRC degradation, RAB12 is thought to play a role in the initiation of autophagy [16]. We speculated that elevation of the GTPase activity and alteration of the subcellular localization of lysosomes in the mutants may have an impact on autophagy. We found a slight increase in relative LC3II protein levels in RAB12 mutants but p62 levels were not affected, indicating a rather minor effect of the RAB12 mutations on activation of autophagy.

Despite RAB12’s important role and plausible link between its function and a neurological disease, screening of additional patient cohorts is warranted to confirm the pathogenicity of RAB12 mutations in dystonia patients. The incomplete segregation in Families C and D as well as the relatively high number (*n* = 83) of carriers of the p.Ile196Val mutation among approximately 140,000 seemingly unaffected individuals in GnomAD are at first glance not compatible with the hypothesis of a pathogenic role of RAB12 mutations in MD. However, it is conceivable that the affected father of Patient D who carries two RAB12 WT alleles and has WD without other neurological symptoms, presents a phenocopy with a different disease cause. Phenocopies are not an infrequent observation, especially in the context of hereditary movement disorders [41]. Furthermore, there is the possibility that the missing disease phenotype in both the mother of Patient D—in contrast to the maternal grandmother - and mother of Family C could be explained by reduced penetrance, a phenomenon often seen in dystonia and other disorders [42]. For instance, penetrance of the GAG deletion in *TOR1A* is reduced to only 30% [43] and 30 presumably unaffected carriers are included in GnomAD (http://gnomad.broadinstitute.org/variant/9-132576340-TCTC-T). Protective genetic variants as well as environmental triggering or other tutelary factors are under discussion in the development of MD [8]. The argument of reduced penetrance can also be applied to the 83 variant carriers in GnomAD. Supporting this idea, having or developing a focal dystonia in the future would not exclude individuals from being included into GnomAD. Similarly, in our about 1000 non-dystonia samples, we also detected only one carrier of a rare missense *RAB12* variant. Of note, the age at onset in our patients was up to 76 years, i.e., late-onset. In theory, developing MD requires extensive training and professional performance in playing an instrument, e.g., in a pianist training especially the fingers of the right hand for ~26,000 h before the average onset of MD [44]. Thus, an accumulating pathological effect (e.g., on TFRC degradation or autophagy) in specific brain regions is conceivable as disease mechanism but cannot be investigated in a cellular model.

Several of our results support the idea of a possible pathogenic role of RAB12 mutations in MD and probably also other dystonias including increased GTPase activities, altered lysosomal distribution, reduced levels of soluble TFRC in patients, and the finding that *RAB12* mutations were found with a higher frequency in dystonia patients than in controls. Of note, *RAB12* seems to be a highly invariable gene. Except for the six listed missense and two synonymous variants (Table 2) we did not detect any additional variant in *RAB12* among the almost 2000 screened individuals. The invariance of the 244-amino acid protein RAB12 is also underlined by the presence of only 55 missense and a single loss-of-function variant in ExAC (http://exac.broadinstitute.org/gene/ENSG00000206418). The ExAC z-score of 1.63 for missense variants indicates that the number of observed variants in *RAB12* was lower than the expected number reflecting decreased tolerance to missense variations in the *RAB12* coding sequence [45]. The intolerance to loss-of-function changes is relatively high with a pLI score (probability of loss-of-function intolerance) of 0.72 [45], which also points to an important functional role of the RAB12 protein in humans. For comparison, in another, similarly sized dystonia-linked protein (THAP1 [THAP domain containing 1], 213 amino acids), ExAC reports a missense z-score of 1.35 and a pLI score of 0.90 while in CDKN1A, a protein of only 164 amino acids and not (yet) linked to any disease, more missense and loss-of-function variants were observed than expected resulting in a lower missense z-score and pLI score, respectively (z = 0.01, pLI = 0.03).

## 5. Conclusions

Taken together, we provide first insights into a possibly pathological function of mutated RAB12 in MD and other forms of dystonia. Screening of more MD patients for mutations in *RAB12* is necessary to confirm *RAB12* as a gene for MD. To shed further light on the pathophysiological mechanism of RAB12, studies on the endogenous level in neuronal cells are warranted.

## Figures and Tables

**Table 1:** Overview of the sequenced patient and control cohorts.

	Next Generation Sequencing	Sanger Sequencing	Gene Panel	Total
**MD patients**	4 ^b^	237	0	241
**Relatives of MD patients**	6 ^a,b^	8	0	14
**Unrelated WD patients**	0	54	20 ^b^	74
**Other dystonia patients**	0	378	226 ^b^	604
**PD patients**	0	0	512	512
**Healthy controls**	0	461	0	461
**Total**	10	1138	758	1906

MD: musician´s dystonia; WD: writer´s dystonia, PD: Parkinson´s disease; ^a^ includes 5 patients with WD; ^b^ All identified mutations were confirmed by Sanger sequencing and all available family members including unaffected were tested for the respective mutation by Sanger sequencing.

**Table 2:** Detected variants in *RAB12* in musician´s dystonia and other dystonia patients as well as in the control group.

Variant (NM_001025300.2, NP_001020471.2)	ID	Polyphen-2 (Score)	Mutation Taster (Score)	MutPred (Score)	CADD Score	Allele Frequency in MD Patients	Allele Frequency in Other Dystonia Patients	Allel Frequency in Controls ^(a)^	Allele Frequency in GnomAD All/non-Finnish EUR
**c.38G>A p.Gly13Asp**	L-3921	Benign (0.086)	Disease causing (0.994)	Benign (0.266)	22.4	1/482	0/492	0/1946	0.0006/n.a.
**c.276A>G p.Arg92= rs372618073**	L-3799 L-5334	n.a.	Disease causing (0.999)	n.a.	8.2	1/482	0/1356	1/1946	0.0003/0.0006
**c.391GA>CT p.Asp131Leu ^(b)^**	L-5385	Benign (0.048)	Disease causing (0.999)	Disease causing (0.492)	25	0/482	0/1356	1/1946	0.00003/0.00006
**c.442G>A p.Ala148Thr**	L-1729	Probably damaging (0.995)	Disease causing (0.999)	Disease causing (0.654)	31	0/482	1/1356	0/1946	n.a.
**c.520G>A p.Ala174Thr rs149427020**	L-11086 L-11092 L-11132	Probably damaging (0.977)	Disease causing (0.999)	Disease causing (0.828)	32	0/482 ^(c)^	0/1184 EUR 3/172 EA	0/1 946 ^(c)^	0.0004/0.0064 ^(d)^
**c.542G>A p.Arg181Gln rs371288995**	L-8527	Probably damaging (0.970)	Disease causing (0.999)	Benign (0.377)	25.3	0/482	1/1356	0/1946	0.00007/0.0001
**c.586A>G p.Ile196Val rs143888944**	L-2381 L-2332 L-3758 L-3781 L-8307 L-9497	Benign (0.323)	Disease causing (0.999)	Disease causing (0.555)	16.5	3/482 ^(e)^	3/1356	0/1946	0.0003/0.0006
**c.693G>A p.Pro231=**	L-5328	n.a.	Disease causing (0.999)	n.a.	10.5	0/482	0/1356	1/1946	0.00001/0.00002

^(a)^ The control population includes 461 healthy individuals and 512 Parkinson’s disease patients; ^(b)^ c.391G>C (p.Asp131His) and c.392A>T (p.Asp131Val) are reported in GnomAD with the exact same minor allele frequency; ^(c)^ Not ethnically matched (patients were of East Asian origin); ^(d)^ Since the variant was exclusively found among East Asian patients, the provided GnomAD frequency is for East Asians instead of Non-Finish Europeans; ^(e)^ In addition, two affected relatives with writer’s dystonia also carried this variant. GnomAD: Genome Aggregation Database at http://gnomad.broadinstitute.org/gene/ENSG00000206418. EUR: Europeans; EA: East Asians; n. a.: not available.

**Table 3:** Clinical findings in *RAB12* mutation carriers.

ID	Variant	Sex	Age at Onset (Years)	Initial Symptom	Age (Years)	Current Symptoms	Family History	Ethnicity
**L-3921**	c.38G>A p.Gly13Asp	male	25	MD	38	MD	negative	German
**L-1729**	c.442G>A p.Ala148Thr	female	76	dervical dystonia	78	torticollis, essential tremor	negative	German
**L-11086**	c.520G>A p.Ala174Thr	male	23	MD	33	task-specific arm dystonia (MD + WD)	negative	South Korean
**L-11092**	c.520G>A p.Ala174Thr	male	17	WD	41	torticollis, WD	negative	South Korean
**L-11132**	c.520G>A p.Ala174Thr	female	58	dystonia of the upper lip	70	torticollis, oromandibular dystonia	negative	South Korean
**L-8527**	c.542G>A p.Arg181Gln	female	58	cervical dystonia	59	torticollis	unknown	German
**L-2381**	c.586A>G p.Ile196Val	male	27	MD	44	MD	positive	German
**L-2332**	c.586A>G p.Ile196Val	male	31	MD	48	MD	negative	Dutch
**L-3781**	c.586A>G p.Ile196Val	male	17	MD	39	MD	positive for dystonia	German
**L-3758**	c.586A>G p.Ile196Val	female	56	cervical dystonia	67	torticollis, shoulder elevation, head tremor	positive for Parkinson´s disease	German
**L-8307**	c.586A>G p.Ile196Val	female	70	cervical dystonia	73	torticollis, head tremor	positive for Parkinson´s disease/tremor	German
**L-9497**	c.586A>G p.Ile196Val	female	41	WD	53	task-specific arm dystonia	positive for Parkinson´s disease/tremor	German

**Table 4:** Results of blood count for members of Family A with the p.Ile196Val mutation.

Parameter (Reference)	Female Patient with WD (L-2276)	Female Patient with WD (L-2283)	Male Patient with MD (L-2381)
Erythrocytes (Mio/µL)	4.5	4.5	4.5
	(4.20–5.40)	(4.20–5.40)	(4.5–5.9)
Hemoglobin (g/dL)	13.6	13.8	14
	(12.0–16.0)	(12.0–16.0)	(14.0–17.5)
Hematocrit (%)	42	40.5	43
	(37.0–47.0)	(37.0–47.0)	(40–53)
Ferritin (ng/mL)	31.2	28.4	276
	(10.0–291.0)	(10.0–291.0)	(18–360)
Transferrin (g/L)	2.4	2.6	2.3
	(2.0–3.8)	(2.0–3.8)	(2.50–3.80)
Fe^2+^ (µg/dL)	75.2	102.6	81
	(50.0–170.0)	(50.0–170.0)	(61.5–156.46)
**Soluble Transferrin receptor (mg/L)**	**0.8**	**0.7**	**1.7**
	**(0.83–1.76)**	**(0.83–1.76)**	**(2.20–4.99)**

## Supplementary Materials

The following are available online at www.mdpi.com/2073-4425/8/10/276/s1, Figure S1: Photograph of Individual L-10289 (mildly affected mother of the index patient from Family D); Figure S2: TFRC colocalized with wildtype and mutant FLAG-RAB12; Figure S3: Lysosomal degradation of the physiological dimeric TFRC was not affected by the RAB12 mutations; Figure S4: Relative LC3-II protein levels are marginally increased in SH-SY5Y cells overexpressing RAB12 Gly13Asp protein and p62 levels remained constant; Figure S5: Original Blots of Figure 3a. Table S1: Demographic data of 604 other dystonia patients tested for mutations in the RAB12 gene; Table S2: Initial NGS variant calls; Table S3: Filter steps of variants generated with recent exome sequencing (Centogene) in Families A, B, and D; Table S4: Primer sequences for Sanger sequencing of RAB12 on DNA level (Exons 1–6), cDNA level, and used for side-directed mutagenesis; Table S5: Variants detected by recent exome sequencing (Centogene) in Families A, B, and D after filtering.
