# Supplementary material for: Functional Characterization of Rare RAB12 Variants and Their Role in Musician’s and Other Dystonias
Source: Genes (Basel). 2017 Oct 18;8(10):276. doi: 10.3390/genes8100276 (PMC5664126; doi:10.3390/genes8100276)
Supplement: Supplementary file 1 [file genes-08-00276-s001.pdf]

Supplementary materials

Functional characterization of rare RAB12 variants and their role in musician's and other dystonias

Eva Hebert et al.

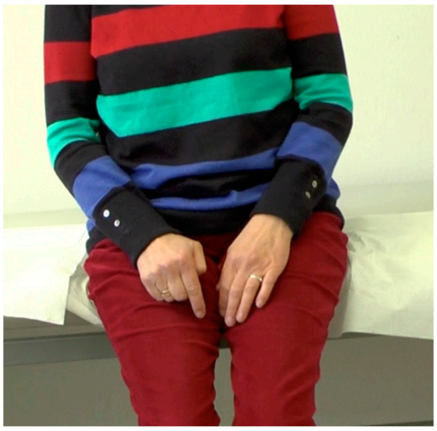

Figure S1. Photograph of Individual L-10289 (mildly affected mother of the index patient from Family D) showing a 15-degree tilt of the trunk to the right as well as dystonic posturing of the right hand (involuntary flexion of the third to fifth finger and thumb and extension of the index finger).

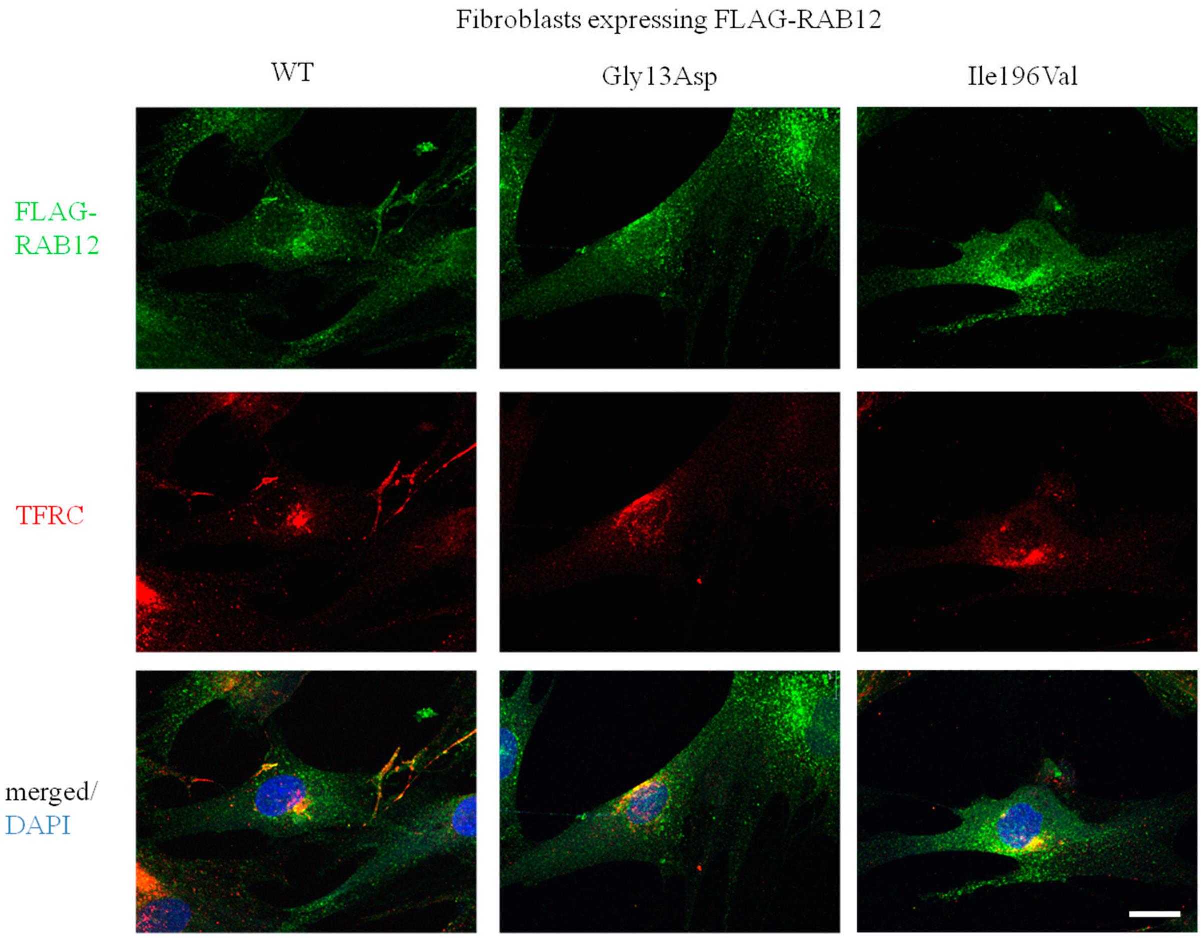

Figure S2. TFRC colocalized with wildtype and mutant FLAG-RAB12. Immunofluorescent staining of fibroblasts expressing FLAG-RAB12 WT, p.Gly13Asp, or p.Ile196Val revealed predominant perinuclear localization of TFRC (red) which overlaps with the localization of FLAG-RAB12 (green) in all three cell lines (WT, p.Gly13Asp, p.Ile196Val). The nucleus was stained with DAPI (blue). Scale bar: 20µm.

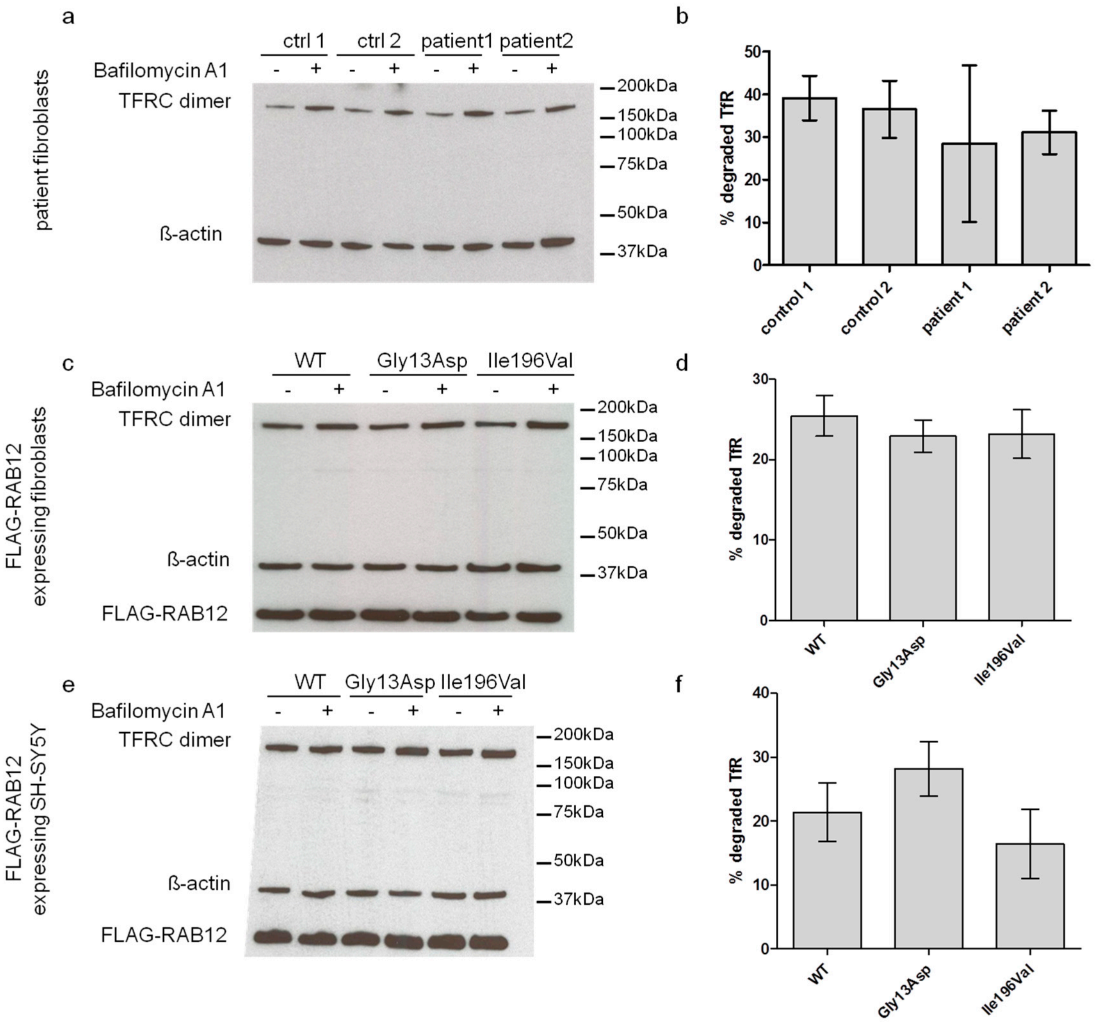

Figure S3. Lysosomal degradation of the physiological dimeric TFRC was not affected by the RAB12 mutations. Western Blot analysis revealed the degradation of TFRC in patient fibroblasts with endogenous expression of RAB12 (a, b) in fibroblasts ectopically expressing FLAG-RAB12 (c, d), and in SH-SY5Y cells ectopically expressing FLAG-RAB12 (e, f). Cells were treated with Bafilomycin A1 for 24h. ß-actin served as loading control and for normalization. Bars in B, D, and F indicate means of three independent experiments ± SEM. ctrl control, WT wildtype

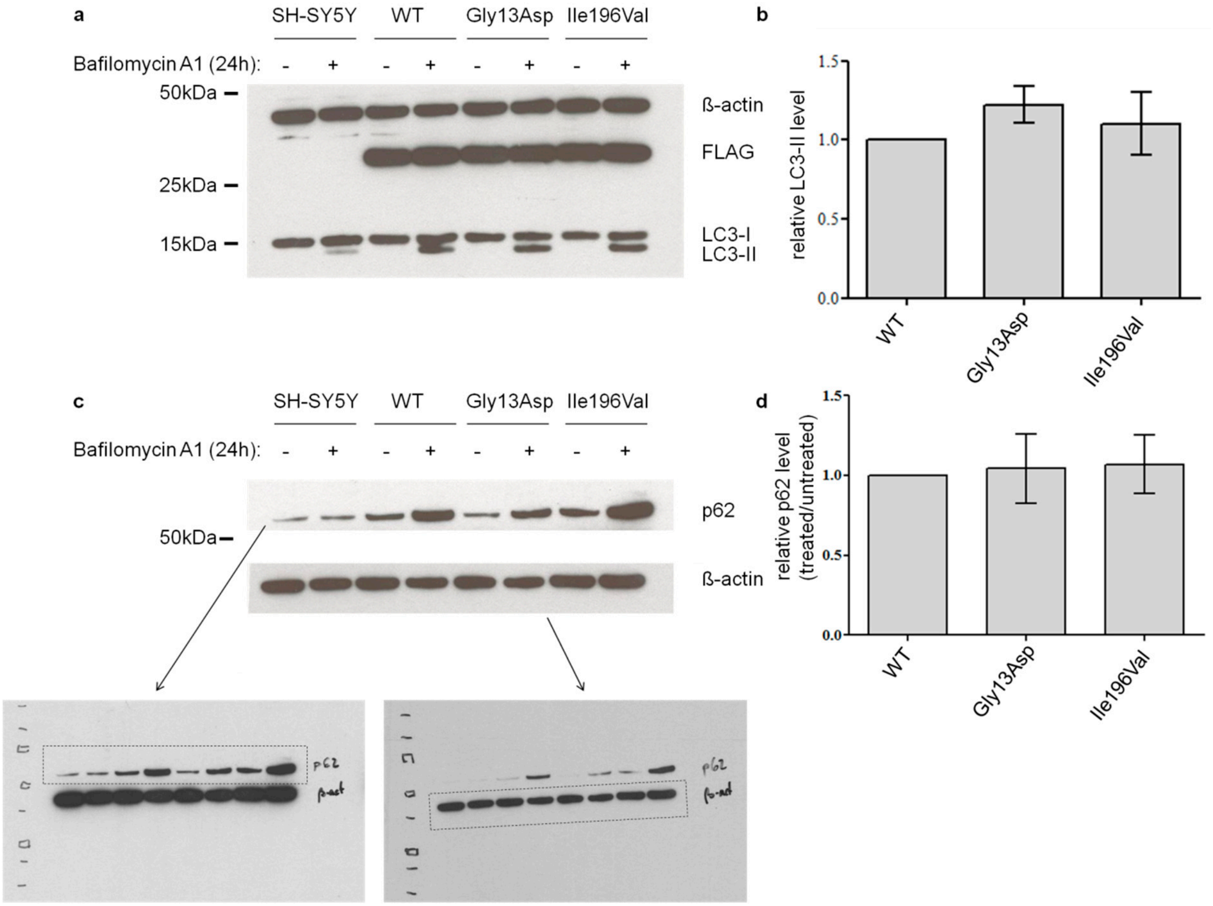

Figure S4. Relative LC3-II protein levels are marginally increased in SH-SY5Y cells overexpressing RAB12 Gly13Asp protein and p62 levels remained constant. a) Western Blot of proteins extracted from stably transfected SH-SY5Y cells. Expression of FLAG-tagged RAB12 WT equals the expression of mutated RAB12 proteins (Gly13Asp, I196Val) (lane 3, 5, 7). Bafilomycin treatment (3nM, 24h) increased the level of active LC3-II proteins whereas the protein levels of RAB12 and ß-actin remained constant. b) Relative LC3-II protein levels are not significantly increased in Bafilomycin-treated RAB12 mutant SH-SY5Y cells (p.Gly13Asp, p.Ile196Val) compared to RAB12 WT expressing cells. n=3 c) Western blot of the same proteins of A) shows that inhibition of autophagy with Bafilomycin A1 causes an elevation of p62 protein levels in the transfected SH-SY5Y cells but not in untransfected SH-SY5Y cells. Protein bands of cropped images (indicated by boxes in the lower blots) were subjected to quantification. Both blots represent the same membrane with same proteins and antibodies, only exposure times differ. D) The ratio of relative p62 in treated vs. untreated cells was not changed in the RAB12 mutants, n=3. Bars indicate means ± SEM.

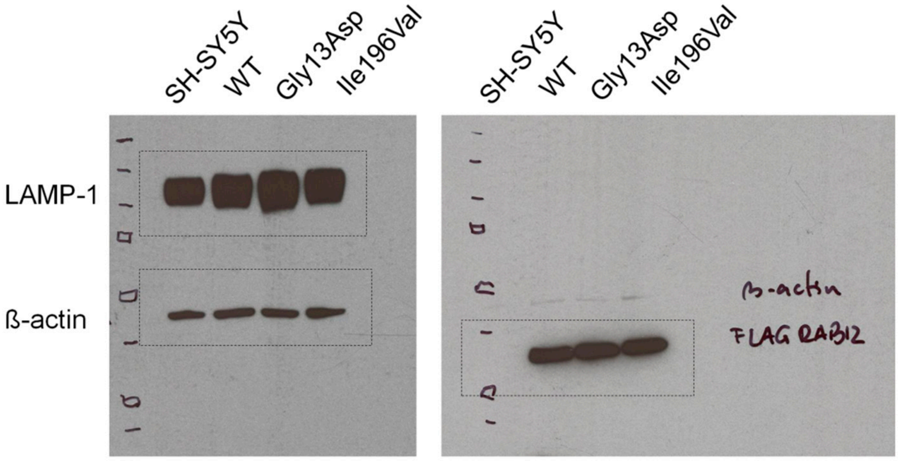

Figure S5. Original Blots of Figure 3A. Boxes around the protein bands indicate cropping lines. The two images were generated from the same membrane. Probing with the FLAG antibody was performed subsequent to LAMP-1 and ß-actin.

Table S1. Demographic data of 604 other dystonia patients tested for mutations in the *RAB12* gene.

Sex	Male	250
	Female	354
Age at onset	Known for	457
	Mean±SD (years)	41±16
	Range	1-82
Family history	Positive	60
	Negative	278
	Unknown	266
Phenotype	Generalized dystonia	59
	Segmental dystonia	123
	Hemidystonia	1
	Multifocal dystonia	3
	Focal dystonia	
	Cranial dystonia	5
	Cervical dystonia	254
	Blepharospasm	56
	Spasmodic dysphonia	6
	Other focal dystonia	92
	Myoclonus-dystonia	5

SD Standard deviation.

Table S2. Initial NGS variant calls. Detected Variants in MD families were filtered to be (1) exonic or splicing, (2) to affect protein function (synonymous variants were disregarded), (3) rare, with a known frequency <1% in the database for single nucleotide polymorphisms (dbSNP132) and (4) shared within definitely affected members within a family.

Family	Code	Variants detected by NGS	Exonic/ splicing variants	Non-synonymous variants	Rare heterozygous variants (frequency <1%)	Same gene locus within one family
A	L-2381	n.a.	28 208	23 753	1 632[a)]	83
	L-2276	n.a.	28 102	23 658	1 498[a)]	
	L-2283	n.a.	27 737	23 357	1 459[a)]	
B	L-2286	3 434 750	20 718	11 000	1 388	509
	L-2322	3 808 124	20 898	10 340	1 044	
C	L-2332	3 815 968	20 583	10 116	1 023	n.a.

[a)] At the time when these exome data were generated and annotated, frequencies from ExAC or 1000g were not yet available and frequencies in the exome variant server at http://evs.gs.washington.edu/EVS/ were not included in the annotated files. Therefore, we initially excluded all variants with an rs number in dbSNP132. Of the remaining variants, 94 variants did overlap between the three affected family members and their frequency was manually looked up in ExAC which identified another 11 variants as common polymorphisms (minor allele frequency >1%).

n.a.: not applicable

Table S3. Filter steps of variants generated with recent exome sequencing (Centogene) in Families A, B, and D.

Variants	Family A	Family B	Family D
all	105480	103152	109082
Found only in affected members, exonic and splice site variants, frequency in ExAC, 1000G, ESP6500 $\leq$1%	844	4279	412
Y Chromosome and mitochondrial DNA excluded	833	4223	412
NGS quality criteria: Frequency $\geq$ 25, read number $\geq$ 20, quality score $\geq$ 100	631	1134	234
All affected members heterozygous	533	477	216
In-house WES: max. 10 homozygous/20 heterozygous carriers in ca. 4500 in-house controls	97	74	131
Synonymous variants excluded	60	46	80

In-house WES: Whole exome sequencing results of 4542 controls were generated at Centogene (Rostock, Germany)

Table S4. Primer sequences for Sanger sequencing of RAB12 on DNA level (Exons 1-6), cDNA level, and used for side-directed mutagenesis.

		Primer sequence (5' -> 3')	Fragment length [bp]
**Exon 1**	F	GATGCTGCTGCCGCTACT	623
	R	CCCATCCCCGAAAGACTC	
**Exon 2**	F	TAGACCGATCACTCAGGATAAG	463
	R	CTCTCTCTTGATGGAAACACTG	
**Exon 3**	F	TCAGGTCAAAGGGAAATAGGG	552
	R	GCAGCTGGATGTAGTGGAATG	
**Exon 4**	F	AACAGCCAAGAGCATTGT	493
	R	TAAAGGAGCACGAAGTCA	
**Exon 5**	F	GAGCGTCAGTGGGACTTTC	351
	R	GCGCCTGAGCACAACTAC	
**Exon 6**	F	AAAGGTCTTCATCTGTGTCA	563
	R	TTGGAGGGAAATAGGTAACT	
**cDNA**	F	CTGCTTCCCTTCCTCCTCT	764
	R	GCCTGGTGATTTCTCTGTCC	
**Mutagenesis**			
c.38G>A	F	CCGCCGCCGTCCCCGGCCC	
p.Gly13Asp	R	GGGCCGGGGACGGCGGCGG	
c.586A>G	F	GGATAACTTCAATGTGGACGAGGTATTTTTGAAACTTGTCGATGA	
p.Ile196Val	R	TCATCGACAAGTTTCAAAAATACCTCGTCCACATTGAAGTTATCC	

Table S5. Variants detected by recent exome sequencing (Centogene) in Families A, B, and D after filtering.

Overlap	Family	Gene	Chrom	Position	Allele	Transcript	coding DNA change	Protein Change	hom/het carriers of total ExAC individuals[a)]	C-scaled CADD score
B+D	B	MUC4	3	195510913	-/AGAGGGG TGGTGTGA CCTGTGG ATACTGAG GAAGCATCGG TGACATGA	NM_018406.6	c.7538_7539ins AGAGGGG TGGTGTGA CCTGTGG ATACTGAG GAAGCATCGG TGACATGA	in-frame	novel	0,018
	D	MUC4	3	195506809	G/T	NM_018406.6	c.11642C>A	p.A3881D	1/10 of 4186[b)]	0,895
A		KIF17	1	20998598	C/T	NM_020816.2	c.2555G>A (rs139517726)	p.R852H	0/79 of 33343	34
		SYTL1	1	27671939	-/A	NM_001193308.1	c.114dupA	frameshift	novel	31
		HFM1	1	91784888	G/T	NM_001017975.3	c.2642C>A (rs369341064)	p.T881N	0/5 of 33333	21,8
		EML6	2	55189686	G/A	NM_001039753.2	c.4996G>A (rs202192837)	p.G1666R	0/30 of 4653[b)]	33
		CFAP65	2	219900226	A/G	NM_194302.3	c.518T>C	p.L173S	0/1 of 33365	13,31
		COPS7B	2	232672281	C/A	NM_022730.1	c.721C>A	p.P241T	0/1 of 652[b)]	23
		GRIP2	3	14581790	G/A	NM_001080423.2	c.317C>T (rs188992337)	p.T106I	1/222 of 32704	15,57
		SLC15A2	3	121641638	G/A	NM_021082.3	c.797G>A	p.R266H	0/2 of 33007	34
		CP	3	148925345	C/T	NM_000096.3	c.841G>A	p.V281I	novel	18,38
		FRYL	4	48581258	T/C	NM_015030.1	c.2260A>G (rs188966880)	p.T754A	1/208 of 32542	4,087
		ANK2	4	114278701	C/G	NM_001148.4	c.8927C>G (rs371343942)	p.S2976C	0/9 of 33361	23,7
		FAT4	4	126373018	C/T	NM_024582.4	c.10847C>T (rs111423173)	p.T3616M	3/388 33343	24,8
		DNAH5	5	13865895	G/A	NM_001369.2	c.4237C>T	p.Q1413*	0/1 of 33194	42
		FNIP1	5	131008129	A/T	NM_133372.2	c.2008T>A	p.L670I	0/1 of 33349	14,76
		ERMARD	6	170155517	A/G	NM_018341.2	c.314A>G (rs542068627)	p.E105G	0/2 of 33320	26,8
		PXDNL	8	52366157	G/A	NM_144651.4	c.1171C>T (rs141730527)	p.R391W	0/111 of 33368	21,6
		CHD7	8	61655009	A/G	NM_017780.3	c.1018A>G (rs41305525)	p.M340V	3/416 of 33358	2,014
		PKHD1L1	8	110477058	G/T	NM_177531.4	c.7997G>T (rs200190153)	p.G2666V	0/37 of 33358	27,6
		PKHD1L1	8	110498978	G/A	NM_177531.4	c.9808G>A (rs200880307)	p.G3270S	0/197 of 33351	19,9
		SLC52A2	8	145583505	C/A	NM_001253815.1	c.353C>A (rs117500243)	p.A118D	1/301 of 33200	21,4
		C8ORF33	8	146278000	C/T	NM_023080.2	c.35C>T (rs139779460)	p.A12V	2/323 of 32872	17,09
		ECM2	9	95277059	C/T	NM_001393.3	c.908G>A (rs143789386)	p.R303Q	2/180 of 66640	11,66
		CARD19	9	95874559	T/C	NM_032310.3	c.424T>C (rs147126180)	p.C142R	1/37 of 4252[b)]	23,6
		PLAU	10	75672059	G/A	NM_002658.3	c.172G>A (rs55744193)	p.G58R	2/582 of 33121	9,936
		LCOR	10	98715216	T/C	NM_001170765.1	c.839T>C (rs200583240)	p.M280T	0/3 of 33336	7,413
		COL17A1	10	105816892	C/T	NM_000494.3	c.1306G>A (rs805697)	p.G436R	2/571 of 33079	10,63
		MUC2	11	1093643	C/T	NM_002457.2	c.5459C>T (rs202186340)	p.T1820M	0/22 of 33085	8,046
		MUC5B	11	1254407	G/A	NM_002458.2	c.2230G>A (rs199581050)	p.G744S	0/122 of 25186	15,04
		MUC5B	11	1268923	G/A	NM_002458.2	c.10813G>A (rs200676815)	p.G3605R	1/100 of 25794	20,1
		MMP26	11	5010915	C/T	NM_021801.3	c.137C>T (rs35365239)	p.S46L	5/579 of 31950	0,657
		MYO7A	11	76870496	G/A	NM_000260.3	c.1007G>A	p.R336H	1/123 of	22,3

Overlap	Family	Gene	Chrom	Position	Allele	Transcript	coding DNA change	Protein Change	hom/het carriers of total ExAC individuals[a)]	C-scaled CADD score
							(rs45629132)		33219	
		PRCP	11	82560132	C/G	NM_199418.2	c.943G>C (rs143734237)	p.A315P	0/57 of 33357	7,56
		MMP20	11	102449808	C/G	NM_004771.3	c.1313G>C (rs61753770)	p.G438A	1/367 of 33348	27,7
		ATM	11	108196837	G/C	NM_000051.3	c.6860G>C (rs1800061)	p.G2287A	0/22 of 33342	10,23
		DLAT	11	111930748	A/G	NM_001931.4	c.1636A>G (rs143152014)	p.T546A	0/33 of 33366	0,003
		ZBTB39	12	57397612	G/C	NM_014830.2	c.1090C>G (rs143767392)	p.R364G	0/24 of 33369	23,4
		MARS	12	57908753	C/T	NM_004990.3	c.2116C>T (rs148501787)	p.R706C	0/27 of 33357	35
		COCH	14	31355389	A/G	NM_001135058.1	c.1348A>G (rs139503327)	p.I450V	0/69 of 33285	22
		SYNE2	14	64593458	C/T	NM_182914.2	c.13850C>T (rs148582250)	p.T4617I	0/63 of 33255	10,88
		PROX2	14	75321867	C/G	NM_001243007.1	c.1747G>C	p.E583Q	novel	28,9
		MLH3	14	75483796	C/T	NM_001040108.1	c.4351G>A (rs28939071)	p.E1451K	0/87 of 33370	23,6
		C14ORF159	14	91639726	C/T	NM_001102368.1	c.550C>T	p.R184W	n.a.	26
		TRIP11	14	92477355	T/C	NM_004239.3	c.1289A>G (rs142579179)	p.E430G	0/25 of 33356	26,6
		ATXN3	14	92537380	-/GCTGCTG CTGCTGCTGC TGCTGCTGC	NM_004993.5	c.890_891ins GCTGCTG CTGCTGCTGC TGCTGCTGC	frameshift	novel	25,7
		ATG2B	14	96772059	T/A	NM_018036.5	c.4600A>T (rs72704878)	p.T1534S	0/301 of 33359	0,004
		TRPM1	15	31294573	G/A	NM_001252020.1	c.4381C>T (rs3784587)	p.R1461C	1/529 of 33369	17,22
		PLA2G4F	15	42437975	C/T	NM_213600.3	c.1661G>A (rs148529696)	p.C554Y	0/203 of 33162	25,7
		GCNT3	15	59911403	A/-	NM_004751.2	c.966del	frameshift	novel	26,6
		FEM1B[c)]	15	68583172	CAAT/GAAC	NM_015322.3	c.1476CAAT>GAAC (rs143637660,rs6494729)	in-frame	2/500 of 33369, 33370/0 of 33370	0,956
		SLC28A1	15	85467271	T/C	NM_004213.3	c.1013T>C (rs139484056)	p.V338A	0/80 of 33365	22,1
		CHSY1	15	101718402	T/A	NM_014918.4	c.1600A>T (rs141305214)	p.I534L	0/16 of 33370	19,03
		TSC2	16	2104355	C/G	NM_000548.3	c.395C>G	p.S132C	novel	14,25
		TSC2	16	2104389	C/G	NM_000548.3	c.429C>G	p.F143L	novel	11,19
		CDH13	16	83378467	C/G	NM_001220488.1	c.778C>G (rs200608482)	p.L260V	0/12 of 33161	23,6
		ZNF469	16	88499661	C/G	NM_001127464.1	c.5699C>G	p.A1900G	novel	12,54
		ABHD15	17	27893566	A/-	NM_198147.2	c.419del	frameshift	1/501 of 30896	29,3
		MTMR4	17	56569151	A/G	NM_004687.4	c.3461T>C	p.I1154T	novel	28,2
		RAB12	18	8636320	A/G	NM_001025300.2	c.586A>G (rs143888944)	p.I196V	0/55 of 32868	16,52
		LAMA5	20	60893686	G/A	NM_005560.4	c.7063C>T	p.Q2355*	novel	38
		VCX	X	7811854	G/A	NM_013452.2	c.418G>A	p.V140M	novel	3,939
Family B		MROH7	1	55119410	A/G	NM_001039464.2	c.811A>G (rs199971430)	p.S271G	0/40 of 33349	4,847
		MYT1L	2	1891354	C/T	NM_015025.2	c.2542G>A	p.A848T	novel	14,78
		EFCAB12	3	129127565	T/C	NM_207307.1	c.1172A>G	p.N391S	0/1 of 9093 [b)]	0,001
		KIF15	3	44816753	G/A	NM_020242.2	c.70G>A (rs146639559)	p.G24S	0/1 of 33280	24,7
		MFN1	3	179080209	C/T	NM_033540.2	c.475C>T (rs575310683)	p.R159C	0/3 of 33363	24,5
		OXNAD1	3	16312496	C/T	NM_138381.3	c.37C>T (rs146704754)	p.R13W	0/14 of 33369	24,9

Overlap	Family	Gene	Chrom	Position	Allele	Transcript	coding DNA change	Protein Change	hom/het carriers of total ExAC individuals[a)]	C-scaled CADD score
		PCCB	3	136019898	C/T	NM_001178014.1	c.971C>T (rs147538201)	p.T324I	0/54 of 33366	24,9
		PPM1M	3	52283798	C/T	NM_144641.3	c.1348C>T (rs142217810)	p.H450Y	0/43 of 32121	9,789
		TRAK1	3	42261019	A/G	NM_001042646.2	c.1997A>G	p.N666S	0/1 of 33370	12,16
		BST1	4	15704847	C/T	NM_004334.2	c.80C>T	p.A27V	0/1 of 2053 [b)]	0,919
		EPHA5	4	66467556	C/T	NM_004439.5	c.713G>A (rs147719164)	p.R238Q	0/10 of 33345	20,9
		RASSF6	4	74477500	C/T	NM_201431.2	c.109G>A	p.A37T	0/1 of 33358	10,88
		ZFYVE16	5	79733022	C/T	NM_001105251.1	c.518C>T	p.P173L	novel	0,067
		ROS1	6	117700302	G/T	NM_002944.2	c.2517C>A (rs199731317)	p.D839E	0/15 of 33231	25,4
		SERINC1	6	122773101	C/A	NM_020755.2	c.691G>T (rs138235986)	p.A231S	0/37 of 33248	25
		PRPS1L1	7	18067266	C/G	NM_175886.2	c.140G>C (rs138696713)	p.S47T	0/5 of 33368	24,1
		SAMD9	7	92731677	T/C	NM_001193307.1	c.3734A>G	p.E1245G	0/4 of 33138	19,51
		SLC29A4	7	5336629	C/G	NM_001040661.1	c.682C>G	p.R228G	novel	32
		FAM83A	8	124204212	T/A	NM_032899.4	c.648+2T>A	splice site	novel	25,3
		IKBKAP	9	111673437	G/A	NM_003640.3	c.1213C>T (rs139703788)	p.R405W	0/58 of 33153	32
		CAPN1	11	64976845	T/C	NM_001198868.1	c.1781T>C (rs148743672)	p.V594A	2/170 of 30235	24,4
		NAALADL1	11	64815499	T/G	NM_005468.2	c.1370A>C (rs139760307)	p.Q457P	0/21 of 31186	23,3
		NARS2	11	78147859	A/G	NM_024678.5	c.1291T>C (rs370150532)	p.Y431H	0/5 of 33334	28,4
		NUP37	12	102494834	A/C	NM_024057.2	c.330T>G	p.D110E	novel	25,1
		LRR1	14	50080993	A/G	NM_152329.3	c.1024A>G (rs186223942)	p.I342V	0/14 of 33079	6,095
		ADAMTSL3	15	84651321	C/T	NM_207517.2	c.2941C>T (rs140552733)	p.R981W	0/230 of 33353	31
		C15ORF41	15	36937461	C/T	NM_001130010.1	c.185C>T (rs139465273)	p.S62L	0/44 of 4549 [b)]	9,025
		DIS3L	15	66618536	G/A	NM_001143688.1	c.2035G>A	p.E679K	0/0 of 33345	23,3
		MAN2A2	15	91454701	C/T	NM_006122.2	c.2030C>T	p.S677L	0/9 of 33295	23,7
		MYO5C [c)]	15	52556426	CACA/TACG	NM_018728.3	c.1008TGTG>CGTA (rs180901348, rs4776032)	in-frame	0/346 of 33301, 33354/9 of 33363	13,97
		RPAP1	15	41822094	A/T	NM_015540.2	c.1027T>A (rs139677678)	p.L343M	0/128 of 32568	24,2
		AMFR	16	56398010	C/T	NM_001144.5	c.1607G>A (rs144358339)	p.R536H	0/36 of 31589	23,5
		DNASE1L2	16	2287265	C/T	NM_001374.2	c.280C>T (rs200934792)	p.R94W	0/58 of 7865 [b)]	31
		SCNN1B	16	23383160	G/A	NM_000336.2	c.1108G>A	p.V370I	0/2 of 33370	0,002
		SLC9A3R2	16	2086440	G/A	NM_001130012.2	c.530G>A	p.R177H	0/11 of 19395 [b)]	26,6
		VWA3A	16	22134476	A/G	NM_173615.3	c.1427A>G (rs200794387)	p.Y476C	0/17 of 16297 [b)]	23,2
		AOC2	17	40997761	C/T	NM_009590.2	c.1118C>T (rs143214815)	p.T373M	0/0 of33370	12,77
		ETV4	17	41606946	C/A	NM_001079675.2	c.1054G>T	p.D352Y	novel	28,8
		ITPKC	19	41223528	C/T	NM_025194.2	c.488C>T	p.P163L	0/8 of 33303	7,44
		MIDN	19	1257143	A/C	NM_177401.4	c.1279A>C	p.S427R	novel	24,4
		RYR1	19	39016132	G/A	NM_000540.2	c.10616G>A (rs143987857)	p.R3539H	1/179 of 32162	26,1
		SBSN	19	36018570	C/A	NM_001166034.1	c.614G>T	p.R205I	novel	10,25

Overlap	Family	Gene	Chrom	Position	Allele	Transcript	coding DNA change	Protein Change	hom/het carriers of total ExAC individuals[a)]	C-scaled CADD score
		TCF3	19	1627375	C/T	NM_003200.3	c.349G>A (rs368163858)	p.V117M	0/2 of 25284 [b)]	22
		CCDC116	22	21991307	A/G	NM_152612.2	c.1790A>G (rs150451119)	p.D597G	0/120 of 32008	6,712
		DEPDC5	22	32206512	G/A	NM_001242896.1	c.1330G>A (rs201394709)	p.G444R	0/68 of 33330	23
Family D		PLCH2 [c)]	1	2431099	AGAA/GGAG	NM_014638.2	c.2601AGAA>GGAG (rs145549953, rs138167803)	in-frame	0/316 of 16001 [b)], 0/313 of 15956 [b)]	2,662
		LUZP1	1	23419368	C/T	NM_001142546.1	c.1387G>A (rs200208053)	p.E463K	0/78 of 33328	25,6
		UBXN11	1	26620735	C/T	NM_183008.2	c.520G>A (rs201526751)	p.E174K	2/155 of 33363	7,387
		MACF1	1	39854002	G/A	NM_012090.5	c.9302G>A (rs185233667)	p.R3101Q	0/43 of 33351	4,803
		SLC44A3	1	95286518	G/C	NM_001114106.2	c.41G>C (rs200690456)	p.G14A	0/6 of 4355 [b)]	0,019
		FAM63A	1	150974740	C/T	NM_001163258.1	c.498G>A (rs142215084)	p.W166*	0/43 of 33361	39
		SCAMP3	1	155230432	G/A	NM_005698.3	c.163C>T (rs11557757)	p.P55S	2/402 of 32949	0,573
		IGSF9	1	159899485	C/T	NM_001135050.1	c.2269G>A (rs200969104)	p.G757S	0/12 of 16661 [b)]	25,2
		IGFN1	1	201166349	A/T	NM_001164586.1	c.271A>T	p.N91Y	novel	20,9
		PQLC3	2	11312136	C/T	NM_152391.3	c.440C>T (rs141308570)	p.A147V	0/2 of 33361	18,66
		TTC7A	2	47238530	G/A	NM_020458.2	c.1348G>A (rs114276698)	p.V450M	2/169 of 32796	25,9
		MYO7B	2	128347710	T/C	NM_001080527.1	c.1898T>C (rs61743282)	p.F633S	0/94 of 17894 [b)]	26,1
		POTEF	2	130832545	C/T	NM_001099771.2	c.2500G>A	p.V834M	novel	25,3
		ANKAR	2	190593470	T/C	NM_144708.3	c.3116T>C (rs150498189)	p.V1039A	0/312 of 33340	21,4
		RAPH1	2	204304816	A/G	NM_213589.1	c.3097T>C	p.S1033P	novel	18,84
		NRP2	2	206590654	C/T	NM_201266.1	c.838C>T (rs79750907)	p.P280S	0/210 of 33364	28,2
		ATIC	2	216182923	C/T	NM_004044.6	c.190C>T	p.R64C	0/1 of 33369	34
		SPHKAP	2	228858308	G/T	NM_001142644.1	c.4663C>A (rs192502309)	p.L1555I	1/227 of 14862 [b)]	20,9
		GAL3ST2	2	242742812	C/T	NM_022134.2	c.428C>T (rs141828605)	p.P143L	0/266 of 30020	32
		RFTN1	3	16411695	C/A	NM_015150.1	c.918G>T (rs199584688)	p.K306N	0/23 of 33370	14,62
		MYH15	3	108174612	G/C	NM_014981.1	c.2293C>G (rs144221103)	p.H765D	0/76 of 33361	24,2
		ZNF639	3	179051533	A/C	NM_016331.1	c.781A>C (rs147750542)	p.I261L	1/113 of 33367	8,338
		SLC2A9	4	9943614	A/G	NM_020041.2	c.737T>C	p.L246P	novel	26,8
		CNOT6L	4	78740530	-/CGCG	NM_144571.2	c.-68_-71dup	5'UTR	novel	5,559
		GPRIN3	4	90171113	G/A	NM_198281.2	c.149C>T (rs147824548)	p.A50V	0/139 of 33359	0,704
		SLC9B2	4	103971435	T/C	NM_178833.4	c.547A>G	p.I183V	0/3 of 33238	0,032
		PDE5A	4	120427058	C/T	NM_001083.3	c.2219G>A (rs139979143)	p.R740K	0/408 of 32595	16,22
		TMEM184C	4	148539204	T/C	NM_018241.2	c.97T>C	p.C33R	novel	21,5
		THBS4	5	79373995	T/-	NM_003248.4	c.2210del	frameshift	novel	35
		NRG2	5	139251390	C/T	NM_013982.2	c.1028G>A (rs545628035)	p.R343Q	0/5 of 33350	28,7
		SQSTM1	5	179250873	G/A	NM_003900.4	c.317G>A	p.R106Q	0/2 of 32188	23,2
		OR2B6	6	27925059	T/C	NM_012367.1	c.41T>C	p.L14P	novel	23
		HLA-G	6	29795822	A/G	NM_002127.5	c.74-2A>G (rs535773328)	splice site	0/45 of 28699	13,77

Overlap	Family	Gene	Chrom	Position	Allele	Transcript	coding DNA change	Protein Change	hom/het carriers of total ExAC individuals[a)]	C-scaled CADD score
		ABHD16A	6	31660879	C/A	NM_021160.2	c.551G>T (rs139667935)	p.R184L	1/154 of 33154	26,3
		HLA-DRB1	6	32549565	G/A	NM_002124.3	c.421C>T	p.H141Y	novel	24,9
		ASCC3	6	101215065	G/A	NM_006828.2	c.1552C>T	p.R518C	0/4 of 33362	29,1
		NFE2L3	7	26224760	G/A	NM_004289.6	c.1442G>A (rs148159120)	p.S481N	1/447 of 33366	0,051
		GLI3	7	42004307	G/A	NM_000168.5	c.4364C>T	p.T1455M	novel	0,006
		SND1	7	127343283	A/G	NM_014390.2	c.746A>G (rs140863632)	p.K249R	0/8 of 33366	16,84
		CSPP1	8	68028252	C/G	NM_024790.6	c.1376C>G (rs146431326)	p.S459C	4/531 of 33369	26,3
		XPA	9	100437758	GTACAA GTCTTACG/-	NM_000380.3	c.772_785del	frameshift	novel	35
		PPRC1	10	103900396	G/T	NM_015062.3	c.2131G>T	p.V711L	novel	0,005
		SYT8	11	1856615	C/T	NM_138567.3	c.226C>T (rs143638495)	p.R76C	0/270 of 29629	11,54
		NADSYN1	11	71196598	G/A	NM_018161.4	c.1466G>A	p.R489Q	novel	33
		WNT11	11	75898191	C/T	NM_004626.2	c.983G>A (rs559115261)	p.R328Q	0/0 of 33039	33
		AAMDC	11	77553582	A/T	NM_024684.2	c.40A>T	p.M14L	novel	18,7
		DDX25	11	125775489	G/A	NM_013264.4	c.172G>A (rs559195926)	p.V58I	0/0 of4514 [b)]	16,52
		FAM90A1	12	8374993	C/T	NM_018088.3	c.820G>A (rs200459059)	p.A274T	0/40 of 32028	0,685
		APOLD1	12	12939898	T/G	NM_001130415.1	c.152T>G	p.F51C	0/0 of 375 [b)]	26,7
		TEP1	14	20869194	G/A	NM_007110.4	c.1498C>T (rs138962979)	p.R500W	0/344 of 33369	34
		NID2	14	52472479	C/T	NM_007361.3	c.4093G>A (rs201207693)	p.A1365T	0/3 of 33332	23,8
		ENTPD5	14	74442714	G/A	NM_001249.2	c.647C>T (rs138322972)	p.T216I	0/96 of 33229	29,6
		NRDE2	14	90756819	G/C	NM_017970.3	c.1975C>G (rs147436404)	p.L659V	0/13 of 33251	16,52
		CHGA	14	93396098	G/A	NM_001275.3	c.293G>A (rs77938104)	p.S98N	3/537 of 33068	17,17
		HERC2	15	28491031	T/C	NM_004667.5	c.3573A>G	p.I1191M	novel	24,8
		EIF3J	15	44829395	-/GGCGGCGGC	NM_003758.2	c.2_3insGGCGGCGGC	in-frame	novel	12,47
		CCDC154	16	1487862	G/A	NM_001143980.1	c.1246C>T (rs571439720)	p.R416W	0/1 of 2226 [b)]	25,2
		NKD1	16	50666241	G/A	NM_033119.4	c.745G>A (rs375061540)	p.V249I	0/20 of 33350	28,4
		P2RX5	17	3594990	G/A	NM_002561.3	c.236C>T (rs142264131)	p.S79L	0/67 of 33293	33
		ALOX15B	17	7950265	A/G	NM_001141.2	c.1328A>G (rs146833910)	p.Q443R	1/292 of 33359	1,021
		TOM1L2	17	17788003	A/G	NM_001082968.1	c.446T>C (rs143220830)	p.V149A	0/106 of 33369	24,7
		CSHL1	17	61987575	C/T	NM_022579.1	c.418G>A	p.D140N	0/1 of 33369	0,334
		AZI1	17	79171966	CA/TG	NM_001009811	c.1418TG>CA	p.V473A	novel[d)]	7,409
		PYCR1	17	79893008	G/A	NM_006907.2	c.334C>T (rs147653673)	p.R112W	1/77 of 29920	23,3
		STARD6	18	51851090	C/T	NM_139171.1	c.635G>A (rs147831274)	p.R212H	0/218 of 33335	12,19
		SERPINB3	18	61322980	C/T	NM_006919.2	c.1084G>A (rs12953909)	p.E362K	2/636 of33351	14,12
		DNMT1	19	10252846	T/C	NM_001130823.1	c.3167A>G	p.N1056S	0/3 of 33267	26,1
		GATAD2A	19	19616186	C/T	NM_017660.3	c.1805C>T	p.A602V	0/7 of 33183	23,4
		FFAR3	19	35850139	G/A	NM_005304.3	c.347G>A	p.S116N	novel	23,4
		HSPB6	19	36247851	G/A	NM_144617.2	c.59C>T (rs11549029)	p.P20L	0/6 of 1816[b)]	19,83

Overlap	Family	Gene	Chrom	Position	Allele	Transcript	coding DNA change	Protein Change	hom/het carriers of total ExAC individuals[a)]	C-scaled CADD score
		BCL3	19	45260434	G/A	NM_005178.4	c.680G>A (rs546909663)	p.S227N	0/5 of 7931[b)]	12,58
		FASTKD5	20	3127441	A/G	NM_021826.4	c.2276T>C (rs147125058)	p.V759A	1/48 of 33099	25,7
		RIN2	20	19955688	A/G	NM_001242581.1	c.1166A>G	p.H389R	0/4 of 32862	7,523
		CST1	20	23731400	T/C	NM_001898.2	c.104A>G	p.Y35C	0/0 of 33360	10,82
		CASS4	20	55028075	C/A	NM_001164116.1	c.1843C>A	p.P615T	novel	24,4
		IL10RB	21	34652167	G/A	NM_000628.4	c.442G>A (rs45545138)	p.V148M	0/128 of 33369	15,82
		UMODL1	21	43519254	G/A	NM_173568.3	c.1150G>A (rs200027906)	p.G384R	0/1 of 33262	21,3
		CRYBB3	22	25597401	C/G	NM_004076.3	c.38C>G (rs147831812)	p.A13G	2/393 of 33214	23,8
		NPTXR	22	39222627	G/A	NM_014293.3	c.976C>T (rs34637063)	p.R326W	2/367 of 33340	34

a) ExAC frequencies were calculated from non-Finnish European individuals.

b) Site is covered in fewer than 80% of the individuals in ExAC, which may indicate a low-quality site.

c) Two synonymous SNVs are reported instead of the listed variant.

d) A synonymous (rs117891655, 0 homozygous and 224 heterozygous carriers among 8415 non-Finnish Europeans) and a missense (rs2659016, 15430 homozygot and 18 heterozygots of 15448 non-Finnish Europeans) SNV are listed in ExAC

n.a. not available
